# Leveraging and exercising caution with ChatGPT and other generative artificial intelligence tools in environmental psychology research

**DOI:** 10.3389/fpsyg.2024.1295275

**Published:** 2024-04-08

**Authors:** Shuai Yuan, Fu Li, Matthew H. E. M. Browning, Mondira Bardhan, Kuiran Zhang, Olivia McAnirlin, Muhammad Mainuddin Patwary, Aaron Reuben

**Affiliations:** ^1^Virtual Reality and Nature Lab, Department of Parks, Recreation and Tourism Management, Clemson University, Clemson, SC, United States; ^2^Environment and Sustainability Research Initiative, Khulna, Bangladesh; ^3^Environmental Science Discipline, Life Science School, Khulna University, Khulna, Bangladesh; ^4^Department of Psychology and Neuroscience, Duke University, Durham, NC, United States

**Keywords:** environmental psychology, generative artificial intelligence, ChatGPT, research methods, research ethics

## Abstract

Generative Artificial Intelligence (GAI) is an emerging and disruptive technology that has attracted considerable interest from researchers and educators across various disciplines. We discuss the relevance and concerns of ChatGPT and other GAI tools in environmental psychology research. We propose three use categories for GAI tools: integrated and contextualized understanding, practical and flexible implementation, and two-way external communication. These categories are exemplified by topics such as the health benefits of green space, theory building, visual simulation, and identifying practical relevance. However, we also highlight the balance of productivity with ethical issues, as well as the need for ethical guidelines, professional training, and changes in the academic performance evaluation systems. We hope this perspective can foster constructive dialogue and responsible practice of GAI tools.

## 1 Introduction

Generative Artificial Intelligence (GAI) has sparked enthusiasm and concern in how we conceive knowledge creation (Nature Editorials, 2023; Stokel-Walker and Van Noorden, 2023). Generated content includes language and text but also images, audio, video, and 3D objects (Supplementary Table S1). Their applications in higher education have raised interest and concerns from educators (Chen et al., 2020), including the UNESCO (Sabzalieva and Valentini, 2023). The applications in scientific writing (Lund et al., 2023) and various scientific domains such as healthcare research (Dahmen et al., 2023; Sallam, 2023), environmental research (Agathokleous et al., 2023; Zhu et al., 2023), and environmental planning and design (Fernberg and Chamberlain, 2023; Wang et al., 2023) have also been discussed. As with all powerful tools, GAI offers opportunities and poses risks depending on the user’s knowledge and choices when using the tool (Dwivedi et al., 2023). For example, ChatGPT’s responses can sound plausible for a research paper but may not match the expected precise and accuracy level for scientific discourse. Therefore, it is time to ask what role GAI can play in environmental psychology. This article aims to prepare the field of environmental psychology to take advantage of GAI while, ideally, avoiding the pitfalls.

Environmental psychology investigates the interaction between human and socio-physical environments, with a practical orientation to solving community-environmental problems using psychological research tools and insights (Stokols, 1978). Given this focus, the field has several challenges that GAI tools might assist. First, human environmental experience depends on the person and the physical, social, and situational contexts (Lazarus and Launier, 1978; Altman and Rogoff, 1987; Hartig, 1993; Wapner and Demick, 2002). Due to the field’s preference for generalized, objective knowledge (Seamon, 1982; Franck, 1987), and researchers’ reliance on existing literature instead of learning from real-life experiences and communities, it struggles to cater to diverse human experiences. Second, environmental psychology’s interdisciplinary nature leads to knowledge gaps for researchers trained in specific disciplines. Researchers may miss advancements in related fields or be unaware of older literature, creating barriers to fully informed research and application (Rapoport, 1997). Third, there can be a lack of alignment between empirical research and practice, especially in environmental planning and design. This may be due to communication gaps (Franck, 1987), lack of integrated perspectives (Hillier et al., 1972), and the complex interactions between factors in practice contexts (Alexander, 1964; Altman and Rogoff, 1987). Fourth, researchers face constraints from the techniques and technologies they can access for applying the appropriate method to a research question. Mastering multiple techniques can be overwhelming and cost-prohibitive, and the opportunity to collaborate, practice, and get feedback from others is not always available.

**Figure 1:**
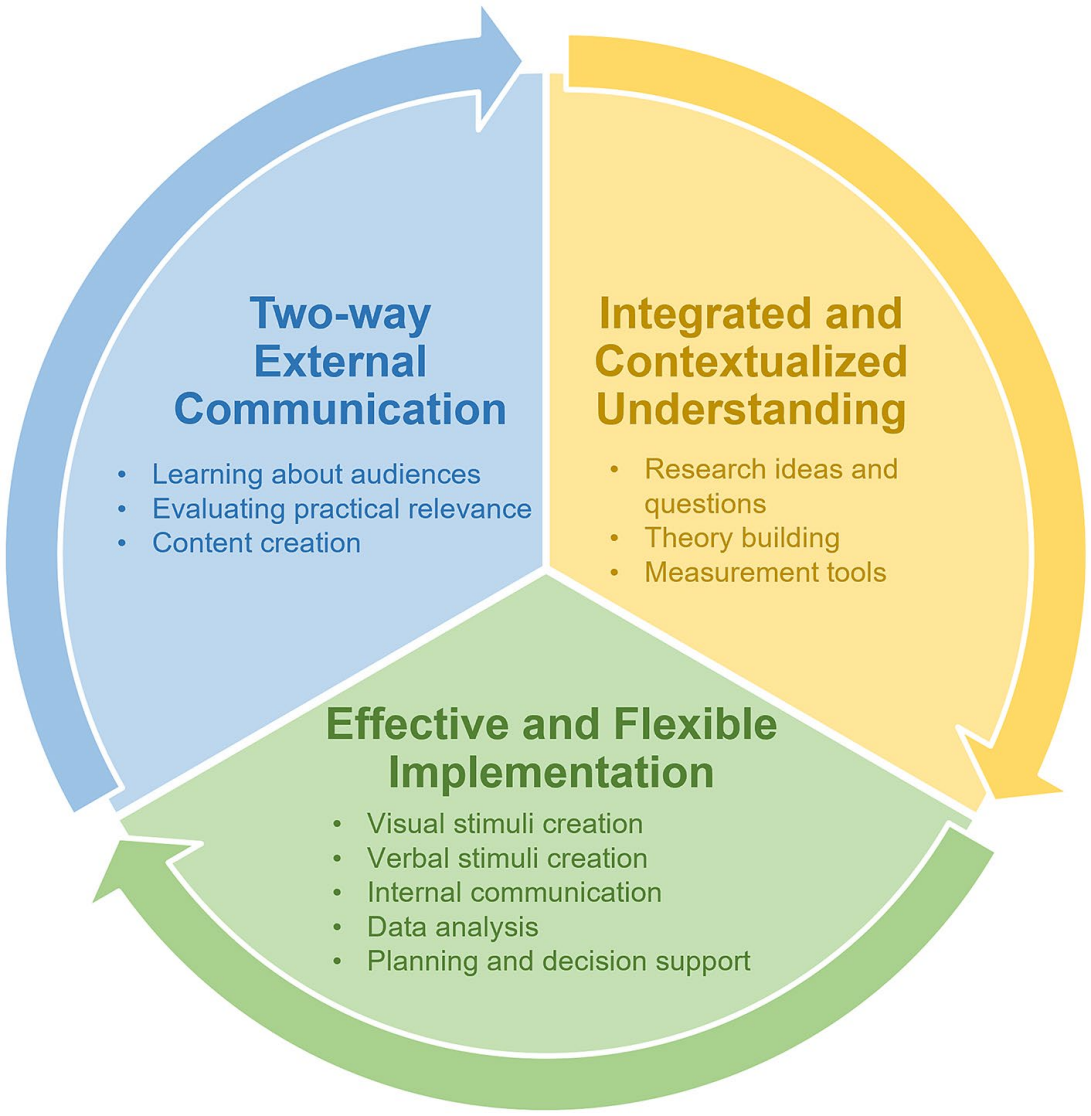
Categories of generative artificial intelligence (GAI) applications for advancing environmental psychology research.

The issues above reflect the limited capacity of human researchers and institutional research structures. We suggest that GAI tools may help fill the various gaps, as they can connect board knowledge to specific contexts, foster interdisciplinary learning through interactive conversations, and assist with various procedures and techniques. To illustrate how GAI can be used to advance environmental psychology as a field, we propose three areas in the research process where GAI tools can be beneficial: (1) integrated and contextualized understanding, (2) effective and flexible implementation, and (3) two-way communication between researchers and external audiences (Figure 1). These three areas cover the various research activities in the research process, such as idea generation, planning and decision support, development of stimuli, data analysis, and communication of findings. To illustrate how these GAI applications could be put into practice, we provide specific examples of our interactions with ChatGPT (see Supplementary material 1–6).

## 2 Integrated and contextualized understanding

Environmental psychology researchers benefit from continual learning, building, and expanding integrated and contextualized understanding of human-environment interaction. Large language models can assist with this pursuit throughout the research process, from brainstorming research ideas, evaluating potential research factors, collecting examples, and interpreting research findings.

### 2.1 Research question formulation

Generative Artificial Intelligence can accelerate the idea-generation process by connecting a loosely described phenomenon or assumption to a list of scientific concepts and theories across different fields. GAI can also help researchers better initiate a study from the perspective of helping to build a knowledge map comprehensively. For example, ChatGPT could assist a researcher interested in human-nature connections by framing questions from the relevant perspectives of psychology, social science, environment, tourism, education, planning, and health science (Ives et al., 2017; Hallsworth et al., 2023). In addition, ChatGPT can provide a matched level of competence to explore unfocused areas (i.e., helping partitioners and researchers to learn other research areas). Therefore, researchers may more likely to identify important but understudied factors and relationships (Supplementary material 1). For example, extreme heat may limit or dissolve how well green space supports healthy behaviors and psychological restoration, though the prevailing modeling has focused on mitigating urban heat islands outside of heat waves (Li et al., 2023).

### 2.2 Theory building

ChatGPT can help perform three theory-building processes described by Walker and Avant (2014), which are derivation (making an analogy and borrowing a concept), synthesis (combining information into a concept), and analysis (breaking down a concept). These processes are more effective when ChatGPT also generates examples and scenarios as hypothetical empirical materials. Researchers can flexibly use ChatGPT’s capacities, including the three strategies we described here. One is an inductive approach, where researchers use ChatGPT to generate examples with social, physical, situational contexts and/or personal factors and abstract and synthesize those examples. This is particularly useful for an initial phase or when empirical data is unavailable. For example, Stokols (2000) has emphasized the research gap in identifying high-impact social-physical circumstances that enhance or constrain human stress coping and functioning. Researchers can use ChatGPT to generate many examples instantly and identify the key factors or groups (Supplementary material 2). Another deductive strategy can be refining and breaking down an existing concept and using hypothetical examples to test the tentative subconcepts. For example, breaking down the concept of compatibility in attention restoration theory (Kaplan, 1995) may help connect it to various environments and human goals (Supplementary material 2). Third but not least, many theory-building approaches involve purposive sample collection to extend or corroborate a theory (i.e., theoretical sampling; Eisenhardt and Graebner, 2007; Corbin and Strauss, 2015). Environmental psychology is often about and can be challenged by everyday experiences. One can use ChatGPT to deliberately collect hypothetical everyday environmental experiences that may contradict a theory. Such counterexamples can be abstracted or synthesized to identify boundary conditions or new mediators and moderators.

### 2.3 Development of measurement tools

Generative Artificial Intelligence tools can ease the item pool generation of psychometric scales and environmental audit tools. One can use ChatGPT to generate items or indicators for a concept or phenomenon that is described broadly or precisely. Alternatively, one can first explore the potential construct structure using the inductive and deductive approaches described in *theory building*, and generate the item pool based on the dimensions. ChatGPT’s large knowledge base and ability to generate many items may help improve content validity. For specific populations or environments (e.g., children, neighborhoods, and urban parks), ChatGPT can also help identify specific factors, assess the suitability of general or related measurement tools, and identify gaps or mismatches. This might improve the relevance of the measurements for the participants and environments. Moreover, ChatGPT’s ability to apply general knowledge and logical reasoning can help explore the potential dimensions of a construct from a list of indicators (Supplementary material 3). By repeating this action, items often categorized into different categories over time may be less valid. Although this cannot replace expert review, it can provide a low-cost check of the quality of ongoing work or existing measurement tools developed using a rational or theoretical approach.

## 3 Effective and flexible implementation

Implementation plays an important role in environmental psychology, but technical issues are a major barrier for researchers implementing their research ideas. GAI can mitigate such barriers and allow researchers to focus more on the research itself. In addition to the implementation aspects common to the social and behavioral sciences, such as institutional review boards (IRBs), recruitment, surveys, interviews, and statistics, environmental psychology has a strong focus on field observation and the creation of environmental stimuli.

### 3.1 Visual stimuli generation

**Figure 2:**
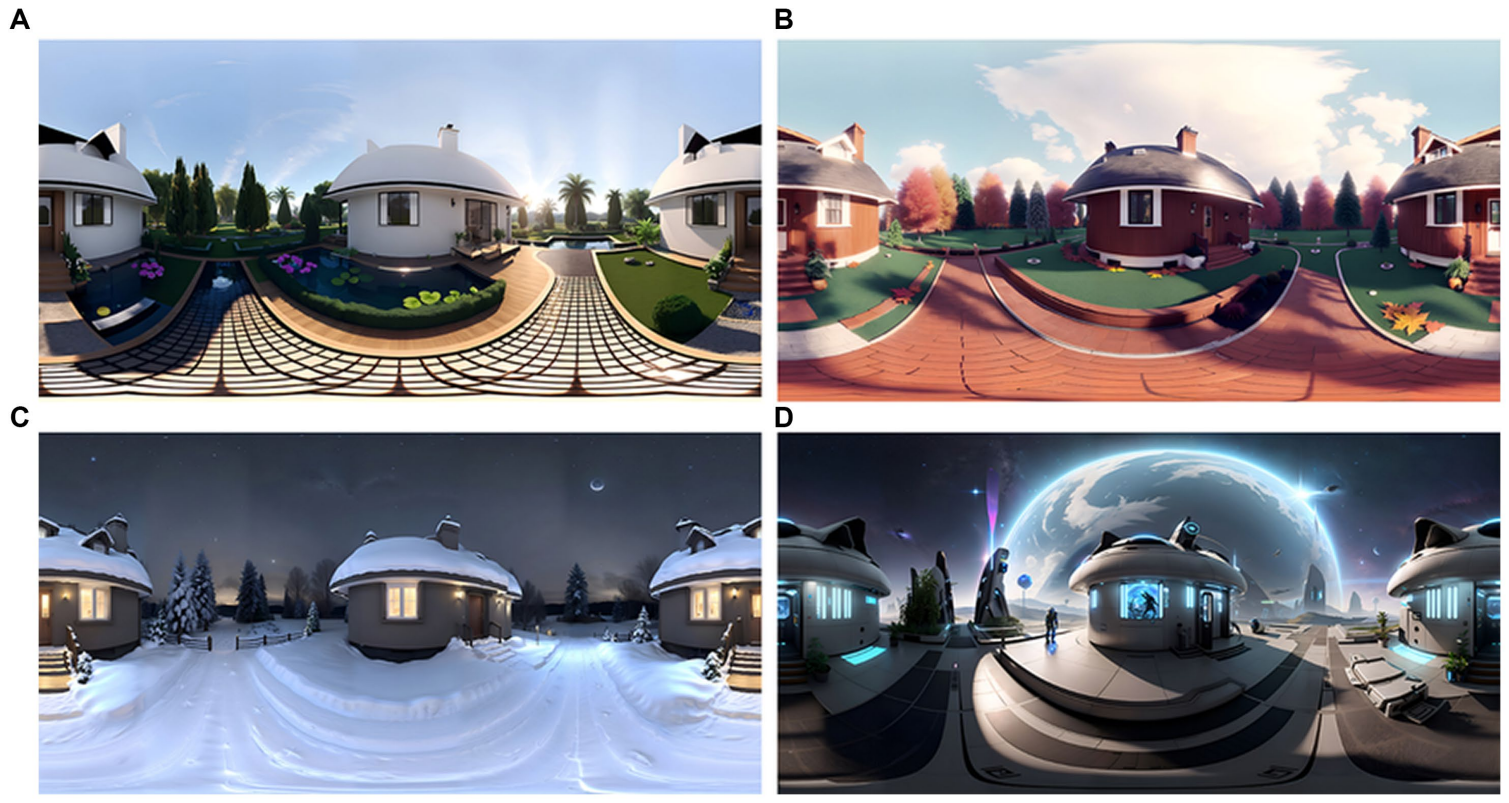
Example of AI generated virtual reality environment form the same structure. Prompts are **(A)** “a summer scene outside a lovely house, realistic”; **(B)** “a fall scene outside a lovely house with leave fall on the ground, realistic”; **(C)** “a winter scene outside a lovely house, with less light and a clear night sky, realistic”; and **(D)** “a space scene outside a lovely house on the moon, Earth in the distance, Sci-Fi.” All variants used the “Mixed Mode” option, which will generate a new image based on the image of the first prompt. Retrieved from Skybox AI, version beta 0.4.2. on May 31, 2023.

Manipulating factors of interest and controlling confounders is crucial for experiment validity. However, learning the required design knowledge and media creation skills can be prohibitive for researchers with non-environmental design backgrounds (Stamps, 2016). Image generators such as DALLE-2, Midjourney, and Stable Diffusion enable rapid generation and modification of imagery to align with the research question (i.e., varying an independent variable in the environment). For example, a Stable Diffusion-based tool, Skybox AI, could generate a VR environment within seconds with a short prompt (Figure 2). Stable Diffusion can also enable style transformation, such as changing the season, time, architectural style and elements while keeping the spatial layout. 3D content generators such as Point E and Shape E can help build virtual 3D Objects. ChatGPT and GitHub Copilot can further help with computer programming tasks in developing virtual scenes in game engines (e.g., Unity 3D or Unreal) for advanced and interactive simulation.

### 3.2 Verbal stimuli generation

Large language models like ChatGPT can create high-quality verbal stimuli with specific contents or factors, communication purposes, and rhetoric considerations. Several research areas, such as decision-making and information-seeking in travel, health, and pro-environmental behavior, could require more complex stimuli beyond visual perception. In this case, researchers can use text generators to create voiceover scripts in the intervention that require specific tones, inclusive languages, and clear narratives. For example, translating scripts for non-native English speakers can lead to misunderstandings and measurement errors when the local dialect is ignored (Solano-Flores, 2006), which could be assisted by GAI. These tools can also create a series of text-based scenarios (vignettes) for priming, contextualizing, and incorporating different parameters for complex conditions (Aguinis and Bradley, 2014). This technique is frequently employed for forecasted scenarios and recreation behavior, pro-environmental behavior, and acceptance of sustainable design (Jaung, 2022; Hu and Shealy, 2023; Urban et al., 2023). For audio narration, advanced text-to-speech tools such as Vall E can mimic tones and emotions for languages, which can be used to test narration scripts, in intervention materials, or as experiment instructions.

### 3.3 Internal communication with participants and institutional review boards

Large language models can help edit language in materials presented to participants. For example, ChatGPT can be used to develop engaging and easily understandable recruitment materials and easy-to-understand experimental instructions. Such language models can also reduce issues such as uncommon or obsolete words, non-inclusive language, and leading questions in existing survey batteries. In verbal-based communication, those tools may improve communication with special populations, such as children, elderly individuals, and people with disabilities and mental health conditions, ensuring understanding, inclusiveness, and compliance. IRB-related documents and materials require specific structures, tones, and an understanding of ethical issues. AI tools might also explain ethical concerns and present potential solutions. It can transfer existing materials into new structures and formats, after cross-checking with specific institutional policies. See Supplementary material 4 for using ChatGPT transferring a description of the procedure from a citation into a training manual, an IRB application, and a consent form.

### 3.4 Basic data analysis

Generative Artificial Intelligence tools can facilitate basic quantitative methods such as the use of R in data cleaning, regression, and factor analysis. ChatGPT can interpret statistical software outputs or model results and suggest visualization options. These tools can assist researchers with qualitative or environmental backgrounds in conducting quantitative or mixed-methods research. Qualitative analysis may benefit less from GAI tools because most qualitative approaches need researchers’ knowledge and experience to interpret data meaning and significance (Creswell and Poth, 2017). However, GAI tools may perform minor roles such as suggesting codes to merge, checking code abstraction levels, and interpreting ambiguous sentences. Also, qualitative data analysis software such as MAXQDA provides a GAI tool^1^ for defining codes based on related quotations. QDA software may also offer an auto-coding function based on regular expression, which is suitable for the exploration of data or more descriptive research. ChatGPT can be used to generate regular expressions to identify objects or features in different texts.

### 3.5 Computer programming

In addition, computational thinking and programming are trending skills for researchers to effectively and flexibly implement data-driven methods and analysis (Chen and Wojcik, 2016). Environmental psychology researchers may increasingly feel they need to learn and apply “advanced” data analysis skill sets, including image, spatial and spatial–temporal data, network data, natural language, or physiological data, in their research. GAI tools excel in assisting the process of program learning and analysis with natural and instant interaction. For example, ChatGPT and GitHub Copilot could help explain code, generate queries, identify relevant coding libraries and functions, explain errors, assisting debug, or write prototype coding for future studies in almost every coding language, such as R, Python, Matlab, etc. (Biswas, 2023; Feng et al., 2023; Rahman and Watanobe, 2023). These capabilities help researchers with both limited and massive coding experience to speed up the research data cleaning, analysis, and visualization process.

### 3.6 Planning and decision support

Successful study implementation requires careful planning, effective problem-solving, and informed decision-making. While support from colleagues, committee members, and advisors is invaluable, such mentors have limited availability. GAI tools’ knowledge base and planning capacities (Wu et al., 2023) can help researchers evaluate methodological choices and trade-offs between them. For example, an environmental psychologist could prompt ChatGPT to generate approaches with higher vs. lower levels of experimental control and ecological validity in a healthcare facility wayfinding study using virtual reality (Joseph et al., 2020). When used in dealing with planning and problem-solving, ChatGPT can facilitate describing an entire procedure and adding details, analyzing the cause of problems, and planning for worst-case scenarios. This can be applied to a large variety of data collection procedures, such as experimental sessions, equipment use, and focus groups. Another example is field observation in urban public spaces (Sussman, 2016). We found that ChatGPT could describe potential iterations in refining observational protocol (Supplementary material 5). It also identified potential issues such as public space being unexpectedly closed, equipment malfunctions or being stolen, failure to adequately familiarize with the site, and notes or data being damaged in transportation. Then one can use ChatGPT to draft a comprehensive logistical checklist and preparation action list.

## 4 Two-way communication between researchers and external audiences

Communicating science with practitioners, policymakers, and the general public requires unique skill sets. This is especially true in environmental psychology, where from its beginnings, this field of research has had an applied emphasis that would benefit from collaboration with practitioners (Wohlwill, 1970; Stokols, 1978, 1995; Devlin, 2018). Communication requires advanced knowledge of external audiences and is not a one-directional stream of information (Ham, 1992). Multi-perspective thinking helps ensure messages are received as intended, and that feedback is considered (Blythe, 2010). GAI tools can assist with this through their large knowledge base that encompasses scientific, professional, and everyday content in addition to the ability to explain audience characteristics, identify relevant scientific concepts, and demonstrate practical relevance.

### 4.1 Learning about audiences and stakeholders

Generative Artificial Intelligence tools can suggest possible expectations and needs of diverse external audiences. Environmental psychology can have implications for designers, planners, healthcare professionals, business owners, residents, land managers, and many other fields. Researchers may be familiar with select professions and communities but are unlikely to consider and know all relevant stakeholders. For example, scholars familiar with speaking with conservation biologists may use an entirely different lexicon about urban greening than scholars trained in public and environmental policy (Vogt, 2018). ChatGPT can be used to identify new stakeholders for research studies, recommend how to communicate with these audiences, learn about their needs and concerns, and provide nuanced “pitches” to capture and maintain stakeholders’ interest (Supplementary material 6).

### 4.2 Evaluating practical relevance

The broad base of procedure knowledge in GAI tools can facilitate identifying relevant practical actions that emerge from research findings. These actions can be useful in suggesting practical implications in scientific publications and practical guidelines. The broad knowledge base can also help assess the feasibility and requirements for a proposed action/approach, provide alternatives, and discuss alignment with practitioner needs, resources, and constraints. These tools can also be used to generate ideas to discuss directly with practitioners. For example, natural sounds may have clear applicability to environmental psychologists interested in restorative landscapes but also have widescale applicability—though perhaps less known to these environmental psychologists—for measuring biodiversity and urban ecology (Fleming et al., 2023).

### 4.3 Communication with the media

In addition to the uses of GAI tools to assist with scientific writing (Buriak et al., 2023; Dwivedi et al., 2023; Lund et al., 2023), these tools can translate scientific writing to social media posts, blogs, storyboards for short videos, technical reports, conference abstracts, and many other content types (Supplementary material 7). The complex concepts and technical language in the original text can be simplified; GAI tools create examples, analogies, and real-world case studies to explain concepts and engage audiences. These tools can also articulate the procedures and steps to implement practical guidance and takeaways from research findings. The text from ChatGPT can emphasize the important implications and limitations for practitioners, policymakers, and other stakeholders and can translate the language into the appropriate tone for a specific audience. For a practical guide on ChatGPT and social media content creation, see Stone (2023).

## 5 Responsible use of GAI in environmental psychology research

Generative Artificial Intelligence tools can have many benefits, but some uses can reduce these benefits or even threaten the value of their research applications. Relying on ChatGPT and other language models for research ideas or a conceptual understanding may compromise originality and critical thinking, or even lead to false information and plagiarism (Beerbaum, 2023; Polonsky and Rotman, 2023; Rahimi and Talebi Bezmin Abadi, 2023; Xames and Shefa, 2023). ChatGPT’s responses can be influenced by how prompts are formulated, such as language ambiguity, complexity level, and clarity of user’s intentions. It is also susceptible to leading questions and can generate misinformation with questions beyond its knowledge base, for example, questions about underrepresented countries and regions. Such complexities of this tool operation can limit the tool’s value. Also, ChatGPT is trained on publicly available datasets. It may copy phrases from the trained documents without giving credit, thus leading to plagiarism. Approximately half of the citations provided by ChatGPT are fabricated, depending on the model version and the type of reference (Bhattacharyya et al., 2023; Walters and Wilder, 2023). Therefore, researchers should always evaluate and fact-check the output to avoid misinformation or plagiarism (Kitamura, 2023). In addition, reliance on GAI in literature searches and idea generation without other sources may constrain creativity (Dwivedi et al., 2023) or impede scholars from developing a foundational knowledge base of a research area.

Reliance on GAI tools to enhance productivity in research can also compromise research validity and the value of research findings. When motivated to publish and seek grants, researchers may use GAI to accelerate their productivity at the expense of originality and professional growth (Dwivedi et al., 2023). While using GAI undeniably boosts productivity, it can bias topic and methodology selection toward those it can assist. These could, in turn, decrease the validity and reliability of research findings, and undercut the benefit of effective and flexible implementation from GAI tools. Furthermore, increased use of GAI for productivity may entrench competition between researchers or institutions regarding publication output, increasing the share of “fast food” publications (van Dalen and Henkens, 2012) with less contribution and significance for understanding human-environment problems.

Therefore, we support the many calls to action from scholars and publishers to create ethical guidelines for GAI use (Dwivedi et al., 2023; Graf and Bernardi, 2023; Liebrenz et al., 2023; Lund et al., 2023; Rahimi and Talebi Bezmin Abadi, 2023). To maintain research originality and significance, it is important to incorporate diverse sources and find a balance between GAI automation and researchers’ thinking. We can refer to the conceptual model of levels and stages of automation from human factor psychology (Parasuraman et al., 2000; Parasuraman and Wickens, 2008), which includes a continuum of automation levels and four automation stages from information-related (information acquisition and information analysis) to action-related (decision selection and action implementation). We recommend using GAI tools for limited stages and at a lower level of automation for the information-related stages. For example, one may select an existing perspective or technique and have ChatGPT facilitate application to new topics (information analysis) or have ChatGPT suggest programming codes and manually test the codes (decision selection). By contrast, using ChatGPT to write a paragraph with references compromises originality (high automation at multiple stages).

Institutions should integrate GAI into professional training. For example, the book by An (2023) provides beginner-friendly guidance on ChatGPT for environmental and health behavior research. Training may also cover advanced skills of GAI, such as prompt engineering, retrieval from files, and fine-tuning. Prompt engineering has been recommended as the initial attempt to obtain a more accurate output (OpenAI, 2024c). It involves improving prompts by providing context, defining expected results, and using intermediate steps for complex tasks (Nori et al., 2023; Wei et al., 2023; White et al., 2023; OpenAI, 2024d). Supplementary material 1, for example, explored the impacts of heat events on greenspace health benefits with an interest in comprehensive, design-oriented answers. Therefore, it set the context of “public health and environmental design” and the expected result structure of “physical, social, and personal contexts.” In addition, uploading files allows the model to retrieve relevant contextual information from a data source outside its training dataset (Retrieval Augmented Generation; OpenAI, 2024a,b,e), which is supported by GPT-4 and the OpenAI API. For example, reference measurement tools may be uploaded in the measurement tool creation example (Supplementary material 3), and a related research proposal can be uploaded for creating field observation protocols (Supplementary material 5). Fine-tuning adapts a model for focused and specialized tasks such as rating, sentiment analysis, FAQs, or generating structured results (Google, 2024; OpenAI, 2024c). This process involves a simplified training process (“tuning”) with specific input and output examples, rather than just supplying instructions (prompt engineering) or unstructured data (retrieval). Thus, it offers the largest level of customization but requires the researcher’s technical capabilities.

We also argue that institutions need to update their policies to promote ethical GAI practices and maintaining academic integrity. Without highlighting the ethical GAI use, scholars and committees involved with tenure and promotion might associate GAI with neutral or negative connotations (i.e., hallucinating false information and biases in training data) instead of their tremendous values to promote scholarship and produce knowledge. Additionally, it is important to recognize topics and methods that are less likely to be promoted by GAI, such as qualitative approaches seeking to deeply understand the lived experiences of individuals and groups. Correspondingly, we see an urgency to modify performance evaluation systems related to publications, funding, and career advancement to protect researchers who use GAI tools in pursuit of increasing the quality and transparency of their research, not only the quantity.

## Data availability statement

The original contributions presented in the study are included in the article/Supplementary material, further inquiries can be directed to the corresponding author.

## Author contributions

SY: Conceptualization, Investigation, Project administration, Visualization, Writing – original draft, Writing – review & editing. FL: Conceptualization, Investigation, Visualization, Writing – original draft, Writing – review & editing. MBr: Conceptualization, Investigation, Project administration, Writing – original draft, Writing – review & editing. MBa: Conceptualization, Investigation, Writing – review & editing. KZ: Conceptualization, Writing – review & editing. OM: Writing – review & editing. MP: Writing – review & editing. AR: Writing – review & editing.

## Footnotes

^1^ https://www.maxqda.com/products/ai-assist
