# Supplementary material for: Leveraging and exercising caution with ChatGPT and other generative artificial intelligence tools in environmental psychology research
Source: Front Psychol. 2024 Apr 8;15:1295275. doi: 10.3389/fpsyg.2024.1295275 (PMC11033305; doi:10.3389/fpsyg.2024.1295275)
Supplement: Supplementary file 1 [file Data_Sheet_1.DOCX]

# Supplementary Data Sheet 1

**Example 1 Explore an unfocused area – health benefits of greenspace in the context of heat event.**

**Custom instructions^[[1]](#footnote-1)^:**

You are a public health and environmental design expert with a non-positivist perspective. You will help me to discover human-environment interactions with physical, social, and personal contexts for a new study topic. Your response is relatively concise.

**Model**

Gpt-4-1106-preview

**USER**

Nature and health research often assume the multiple health benefits of green spaces. However, contextual factors may limit or dissolve how well green space supports healthy behaviors and psychological restoration. Tell me 10 different factors.

**ASSISTANT**

Certainly! Here are 10 contextual factors that can impact how green spaces support healthy behaviors and psychological restoration:

1. Accessibility: If green spaces are difficult to reach due to distance, inadequate transportation, or poor connectivity, their health benefits could be less accessible to certain populations.
2. Safety: Perceived and actual safety concerns, such as crime or dangerous wildlife, can discourage individuals from using green spaces, thus limiting their positive health impacts.
3. Maintenance: Poorly maintained green spaces with litter, overgrown paths, or damaged facilities can reduce their attractiveness for physical activity and mental restoration.
4. Cultural Relevance: Green spaces that do not resonate with the cultural practices and preferences of the local population may be underutilized and less likely to contribute to well-being.
5. Social Inclusivity: Spaces that are not designed to be inclusive of various age groups, abilities, and socio-economic backgrounds may not support community health equitably.
6. Design Quality: The layout, types of vegetation, availability of amenities, and aesthetic appeal can influence how people interact with green spaces and experience their benefits.
7. Biodiversity: Green spaces with low ecological variety may offer reduced opportunities for interaction with nature, potentially impacting the restorative effects they provide.
8. Noise and Air Quality: Pollution from nearby traffic or industrial activities can impair the restorative experience of urban green spaces and discourage physical activities.
9. User Capacity: Overcrowded green spaces can lead to competition for resources, lack of tranquility, and reduced opportunities for personal restoration and relaxation.
10. Climate and Weather: Extreme weather conditions, such as excessive heat, cold, or precipitation, can limit the use and enjoyment of outdoor green spaces and the associated health benefits.

These factors underscore the complexity of human-environment interactions and highlight the importance of a context-sensitive approach in urban planning and public health to maximize the benefits of green spaces for all community members.

**USER**

Great, let's focus on heat events. How can heat events moderate greenspace benefits? Give me 5+ reasons, and use the other contextual factors if applicable; do not use them if not applicable.

**ASSISTANT**

Heat events can notably moderate the benefits of green spaces due to several interacting factors:

1. Reduced Physical Activity: High temperatures can discourage outdoor exercise and movement, as people may find it uncomfortable or even dangerous to engage in physical activity under extreme heat conditions, leading to a decrease in usage of green spaces for healthful activity.
2. Thermal Comfort: Green spaces without adequate shade, whether from trees or man-made structures, can become heat islands themselves, exacerbating the discomfort during heat events. If visitors do not find these areas thermally comfortable, they are less likely to perceive them as restorative or beneficial for their wellbeing.
3. Dehydration and Heat Illness: Prolonged exposure to high temperatures without proper hydration facilities (like water fountains) and shaded areas can increase the risk of heat-related illnesses, such as heatstroke or dehydration, which can negate the health benefits associated with green spaces.
4. Altered Timing of Use: Heat events might limit the use of green spaces to cooler early morning and evening hours. This shift in timing can affect accessibility—particularly for individuals with inflexible work schedules or caregiving responsibilities—and reduce overall use and associated health benefits.
5. Impact on Vulnerable Populations: The elderly, young children, and individuals with preexisting health conditions are more susceptible to adverse effects from extreme heat. These groups might be particularly disadvantaged during heat events, as they may need to avoid exposure to high temperatures, thus missing out on the benefits of green spaces.
6. Deterioration of Environmental Quality: Prolonged heat events can stress vegetation, leading to wilting or dying plants, which can diminish the aesthetic and ecological quality of greenspaces. This may, in turn, influence individuals’ desire to visit and spend time in these areas, reducing opportunities for stress reduction and psychological restoration.

These reasons highlight the necessity for heat-mitigation strategies in the design and management of urban green spaces, such as incorporating shaded areas, water features, and cooling infrastructure to ensure that green spaces remain beneficial and accessible during periods of extreme heat.

**USER**

I appreciate you mentioned design factors like shade from trees and the work schedules of users. Now I want to better explore the latter. What could be some types of people's use patterns (e.g., not in routine, in daily routine, what daily routine) that are related to the impact of heat on greenspace benefits?

**ASSISTANT**

Heat events can influence different types of green space use patterns, each of which relates to the daily routines and lifestyle choices of individuals. Here are several examples:

1. **Recreational Use Outside of Routine:** Individuals who visit green spaces sporadically for leisure or recreation may be more likely to forego these visits during heat events. Their non-routine use means they have flexibility and may choose cooler conditions for activities like picnics, sports, or family outings.
2. **Incorporated into Daily Routine for Exercise:** Some people have a routine of visiting green spaces for jogging, walking, or cycling, typically during specific times such as early morning or evening. During heat events, they might either shift their activities to even cooler times or temporarily pause their outdoor exercise routines.
3. **Commute Through Green Spaces:** For those who utilize green spaces as part of their daily commute, extreme heat could significantly disrupt their routines. Commuters may seek alternative, possibly less scenic but cooler indoor routes, losing the psychological benefits of a green commute.
4. **Scheduled Group Activities:** Group fitness classes, community gardening, or educational programs that routinely take place in green spaces might face cancellations or reduced participation during heat events. Participants who rely on these scheduled activities for social interaction and physical activity might then miss out on these benefits.
5. **Lunch Breaks and Brief Workday Escapes:** Office workers and nearby employees who customarily use green spaces for breaks during the workday might opt to stay indoors in climate-controlled settings, thereby forgoing the mental restoration that brief nature interactions usually provide.
6. **Childhood Play and Development:** Children's play patterns in green spaces, which are often routine and critical for development, might be adjusted, with parents choosing indoor playgrounds or water-based activities to keep children cool, at the cost of more diverse interaction with nature.
7. **Shift Workers and Non-Standard Hours:** People working non-standard shifts may have routines that do not align with the cooler parts of the day, making it more challenging for them to utilize green spaces for restorative purposes during a heat event.

People's various engagements with green spaces during routine and non-routine activities are significant indicators of how heat events can modulate the health benefits of these environments. Understanding use patterns can inform urban planning measures, such as ensuring there are shaded paths for commuters, misting stations for exercisers, and climate-resilient playgrounds for children, to mitigate the impact of heat events on green space utilization and benefits.

**USER**

These types of engagements are insightful and relevant, and we need to further abstract/classify them to align with the impact of heat events, in a way like qualitative coding or theory building. Give me three classification schemes and explain their pros and cons.

**ASSISTANT**

Certainly, classifying green space use patterns regarding heat events can help create a framework for better understanding and mitigating the negative impacts of heat on user engagement. Here are three classification schemes with their advantages and disadvantages:

**1. Time-Based Classification Scheme:**

*Classification:*

- Peak Hour users: Those who use green spaces during the hottest parts of the day.
- Off-Peak Hour users: Those who use green spaces during cooler morning or evening hours.

*Pros:*

- Aligns with the diurnal pattern of heat variation, offering clear guidelines for designing heat mitigation strategies, such as targeted shading or water features for peak hours.
- Simplifies scheduling for maintenance and programming around user density to minimize heat exposure.

*Cons:*

- Overlooks the diversity of user needs and preferences that might not align neatly with time-based categories.
- May not accurately reflect seasonal or weather-related variations in usage.

**2. Activity-Based Classification Scheme:**

*Classification:*

- Active users (engaging in physical activities like sports and exercise)
- Passive users (participating in activities like reading, picnicking, or meditating)
- Transitional users (using green spaces as routes for commuting or walking between destinations)

*Pros:*

- Clearly differentiates between types of use, allowing for tailored design elements (e.g., more shade for passive areas, cooling stations near active zones).
- Activity-based distinctions can inform the development of programs and facilities to support diverse uses during heat events.

*Cons:*

- The boundaries between these categories can be fluid, with one individual's activities potentially spanning multiple classifications.
- Does not consider the social and cultural context that might influence activity decision-making during heat events.

**3. Demographic-Based Classification Scheme:**

*Classification:*

- Vulnerable Populations (elderly, children, those with health issues)
- Work-Oriented users (people using green spaces during work breaks or for commuting)
- Recreational users (individuals seeking leisure and social interaction)

*Pros:*

- Addresses the specific needs and vulnerabilities of different population groups, facilitating targeted interventions (e.g., setting up cooling centers for vulnerable populations).
- Supports demographic-focused public health campaigns and urban design to ensure inclusive access to green space benefits during heat events.

*Cons:*

- Can be reductionist, failing to capture the complexity of individuals' identities and how they interact with green spaces (e.g., an elderly person may also be a recreational users).
- Demographic data may be difficult to obtain, and the assumption based on demographics may lead to stereotyping and ineffective solutions.

Each classification scheme provides a lens through which to examine and design for the impact of heat on green space benefits. While they all offer valuable insights, combining elements from each approach could offer a more comprehensive understanding of user patterns and contribute to more effective, inclusive urban planning and public health strategies.

**USER**

I like the complexity in your answer and that you brought up the implication of classification on design intervention and public health campaigns. From what you said, I think peak hour, personal vulnerability, and flexibility in adjusting activities seem to be the key. However, please evaluate my understanding and let me know what I have missed from your previous responses.

**ASSISTANT**

Your understanding synthesizes key aspects from the classification schemes and their relation to the design and public health implications regarding heat events in green spaces. Let's evaluate and expand on the points you've highlighted:

1. **Peak Hour users:**
   - *Understanding*: You've correctly identified that considering peak hour usage is important because heat events tend to be more intense during certain times of the day. This understanding is crucial for planning interventions like creating shaded areas or scheduling activities during cooler times.
   - *Potential Miss*: A consideration that might be missing is the off-peak hour users and their patterns, which also require attention to ensure the green spaces are accessible and beneficial outside peak hours.
2. **Personal Vulnerability**:
   - *Understanding*: Acknowledging personal vulnerability due to age, health status, or other factors is indeed critical, as these aspects determine the extent to which individuals can cope with heat and utilize green spaces safely.
   - *Potential Miss*: It's vital to not only focus on those traditionally viewed as vulnerable but also consider individuals who might be unexpectedly affected by heat events, such as athletes or outdoor workers, who may also require specific interventions.
3. **Flexibility in Adjusting Activities**:
   - *Understanding*: Recognizing that individuals have varying degrees of flexibility in adjusting their activities in response to heat events is essential for creating inclusive spaces. This flexibility influences when and how people can use green spaces.
   - *Potential Miss*: In your assessment, consider the systemic and institutional factors that impact flexibility, such as work policies, school schedules, or public transit availability, which can enable or constrain individuals' capacity to adjust their routines.

Your understanding touches on important aspects of how heat events can impact green space benefits. However, additional nuances could be considered:

- **Intersecting Factors**: How time-based, activity-based, and demographic-based factors intersect and influence each other might need further exploration to fully grasp the complexity of heat events' impacts.
- **Cultural and Social Contexts**: The role of cultural and social norms in shaping how individuals perceive and respond to heat, which can vary significantly across different communities.
- **Environmental and Urban Design**: The broader environmental context, including urban design features like built infrastructure, greenery density, and water elements, all contribute to the thermal experience of green spaces and their capacity to provide cooling and comfort during heat events.

Overall, your reflection captures substantial elements of the discussion, and enriching it with the considerations of intersectionality, social context, and environmental design would offer a more holistic perspective.

**USER**

With a background in urban design, I could not imagine how specific cultural and social contexts shape how people perceive and respond to heat. To help me make sense, can you give me several community examples in a personal narrative format?

**ASSISTANT**

Certainly! Here are several examples in narrative format that illustrate how cultural and social contexts can shape the perception and response to heat within different communities:

**Community A - The Urban Gardeners:** In the heart of a bustling city, there's a community with a strong tradition of urban gardening. Local residents, many from a culture that deeply values connection to the land, eagerly wait for the weekends to tend to their communal plots. However, during heatwaves, the communal gardens become desolate, the intense sun beating down on exposed soil. Initially, the gardeners, accustomed to the heat due to their heritage, try to adhere to their routine, wearing wide-brimmed hats and loose clothing. Gradually, they adjust their schedule to the cooler early mornings, bringing the community together to share stories and coffee as they work. This shift honors their cultural ties to the land while respecting the new rhythms imposed by the climate.

**Community B - The Riverside Retreat:** Downstream, a river winds through a neighborhood where the elderly population frequently congregates by the water's edge. Here, cultural norms dictate that the river is a place of social gathering and relaxation. When a heat event strikes, the community council erects temporary awnings and misting stations along the riverside to offer relief from the relentless heat. The elders continue to visit, as their daily conversations and card games by the river are more than a pastime; they're a critical ritual for wellbeing. This adaptive response preserves social structures while integrating necessary modifications for comfort and health.

**Community C - The Midday Sports League:** In another district, a vibrant sports league unites the youth after school. The culture of this community places a high value on athletic prowess and competition. Heat events pose a significant risk for these activities; however, instead of canceling games, the league institutes heat safety protocols drawing upon the community's respect for sportsmanship and discipline. Coaches teach players about hydration and heatstroke prevention, and games are rescheduled to the evening when the temperature drops—a practical solution that upholds the community's sense of identity and values around sport.

**Community D - The Festival Town:** Seasonal festivals are the pride of a small town where the cultural identity revolves around yearly celebrations that draw crowds to outdoor marketplaces and performances. As temperatures rise, festival planners incorporate heat-conscious strategies without diluting the essence of their traditions. Cooling tents become new meeting spots, and performance schedules adapt to feature evening shows. Even the cuisine shifts, with vendors offering more refreshing, hydrating options. These adaptations show the community's resilience, maintaining the joy of festivals while responding innovatively to weather challenges.

These narratives demonstrate that cultural and social contexts play a pivotal role in how communities perceive and manage the impacts of heat events on green space usage. The responses are a tapestry of adjustments that honor traditions and collective values while ensuring safety and comfort during challenging climatic conditions.

**USER**

Great, are there any Cultural and Social Contexts that impede the effective response to heat events on green space usage? Also, please use one person (like "Sam") as the narrator of the story, not multiple people (like "coaches").

**ASSISTANT**

Certainly. Here's a narrative from the perspective of Sam, illustrating how certain cultural and social contexts might impede an effective response to heat events on green space usage:

Sam's Story: Overcoming Cultural Barriers to Beat the Heat

My name is Sam, and I work as a community liaison in a vibrant, close-knit neighborhood that has been my home since childhood. The area is known for its beautiful parks and green spaces, which serve as central hubs for gatherings, celebrations, and daily life. However, our strong cultural ties to outdoor living sometimes clash with the realities of increasingly frequent heat events.

In our community, social standing is often associated with endurance and resilience, qualities celebrated in stories and customs. Cancelling or adjusting outdoor events due to heat is not in our nature. During the hottest days, rather than seeking shelter and cooler indoor activities, people often push themselves to maintain normalcy, leading to heat exhaustion incidents.

Once, at the height of summer, we had a street fair that had been planned for months. The heat was unbearable, yet the fair went ahead as scheduled. It's seen as dishonorable to complain about the heat or to admit that it's too much to bear. As the day wore on, several attendees suffered from heat-related illnesses, and the event was reluctantly cut short.

I remember sitting with my neighbor, an elderly man revered for his stoicism, as he confided in me his concern about the younger generation facing the heat but refusing to speak up due to cultural expectations. He said, "Sam, we need to change, but it's hard when traditions run so deep."

Since that wake-up call, I've worked to shift perspectives by incorporating heat safety into our cultural narrative. It's not about taking away from our customs but about adapting them. I started organizing early morning community walks, explaining that we respect the sun's power by enjoying the cool of dawn. We introduced shaded areas in our parks, equipped with water misters, subtly integrating them without altering the landscape's familiar feel.

It's a challenge to balance cultural identity with the realities of a changing climate, especially when those identities are rooted in utilizing outdoor spaces. But I've witnessed how individuals like my neighbor, whose words carry weight, can be catalysts for positive change when they start shifting the conversation.

Through persistent outreach and respectful dialogue, we're learning that adapting to heat events isn't a sign of weakness; it's a testament to our community's intelligence and ability to thrive in the face of adversity. It's about ensuring that our green spaces remain centers of health and connection, no matter the weather.

**USER**

Great explanation. Let's go back to the factors that moderate the impact of heat on GS benefits. We have peak hours, personal vulnerability, and flexibility in adjusting activities as personal factors. What about the environmental factors that lessen or worsen the impact of heat on GS benefits? Please respond with two scales: park/site and a larger scale. I mentioned the latter because the availability of different types of health behavior resources (GS, non-GS, physical, social, etc.) can make a difference.

**ASSISTANT**

Certainly, environmental factors at both the park/site scale and the larger urban or regional scale can influence the impact of heat on the benefits derived from green spaces (GS). Here are some of these factors at both scales:

**Park/Site Scale:**

1. **Shade Provision:**
   - **Lessens Impact:** Adequate shade from trees or built structures like pergolas within the green space can provide cooler microclimates, allowing individuals to engage in outdoor activities even during hot weather.
   - **Worsens Impact:** Insufficient shade can lead to greater heat exposure, increasing discomfort and health risks for users, thereby reducing the space's usage and benefits.
2. **Water Features:**
   - **Lessens Impact:** Features like fountains, streams, and spray parks can help cool the air and users, making the green space more appealing and usable during heat events.
   - **Worsens Impact:** Absence of water features may limit the cooling effects within a green space, potentially deterring use when temperatures are high.
3. **Material and Design Choices:**
   - **Lessens Impact:** Using light-colored, reflective materials for pathways and incorporating vegetation can reduce heat absorption. Thoughtful landscaping that maximizes airflow can keep spaces cooler.
   - **Worsens Impact:** Dark, heat-absorbing materials and design choices that block breezes can increase the local temperature, diminishing the park's restorative qualities during heat events.

**Larger Scale (Urban/Regional):**

1. **Green Infrastructure and Connectivity:**
   - **Lessens Impact:** A network of connected green infrastructures, such as green roofs, street trees, and urban corridors, can contribute to lower urban temperatures, making it more comfortable to move between and use green spaces.
   - **Worsens Impact:** Fragmented green infrastructure can lead to isolated hot spots and reduced mobility for individuals seeking out green spaces for respite.
2. **Urban Planning and Land Use:**
   - **Lessens Impact:** Strategic urban planning that includes diverse types of health behavior resources, including accessible green spaces interspersed with shaded transit routes and community centers, can offer more options for maintaining health behaviors during heat events.
   - **Worsens Impact:** Lack of comprehensive planning can result in limited access to cool spaces or amenities, forcing individuals to spend more time in heat-stressed environments.
3. **Availability of Cooling Facilities:**
   - **Lessens Impact:** Cooling centers and public buildings with air conditioning can provide safe havens during extreme heat, offering supplemental spaces for physical and social activities when outdoor conditions are hazardous.
   - **Worsens Impact:** Inadequate provision of public cooling facilities may leave residents with no alternative but to either endure the heat or remain isolated indoors.

Understanding these environmental factors at both the park/site level and the larger scale is crucial for mitigating the adverse effects of heat on the health benefits of green spaces. It ensures that the design and planning of these spaces and their wider context respond dynamically to the challenges posed by heat events, promoting healthy behaviors and psychological comfort.

**USER**

Great, can we summarize the personal, social, and environmental (physical) factors into three tables (each type in a table), with the first column being the name of the factor and the second column briefly explaining the impact?

**ASSISTANT**

Absolutely! Below are the tables summarizing the factors according to their type:

**Table 1: Personal Factors**

| **Factor** | **Impact on Heat Events and Green Space Benefits** |
| --- | --- |
| Peak Hour Usage | Engagement patterns during the hottest parts of the day can lead to decreased use due to increased discomfort and health risks. |
| Personal Vulnerability | Individuals with greater vulnerability (e.g., elderly, young, those with health issues) are more susceptible to heat and may require additional accommodations or avoid green spaces during heat events. |
| Flexibility in Adjusting Activities | The ability to adapt activity timing (earlier or later in the day) or type (less strenuous) in response to heat can help maintain green space usage and derived benefits. |

**Table 2: Social and Cultural Factors**

| **Factor** | **Impact on Heat Events and Green Space Benefits** |
| --- | --- |
| Cultural Attitudes | Culturally ingrained perceptions or behaviors, such as a strong outdoor lifestyle or stoicism in the face of discomfort, can lead to continued use despite risks or, conversely, to adaptive changes that balance tradition with health. |
| Social Norms and Community Practices | Established community behaviors and expectations may encourage or discourage use during heat events; social support networks can either offer or fail to offer guidance for safe usage. |
| Public Awareness and Education | The level of community awareness and knowledge about heat risks and safety can influence how people modify their behaviors to effectively utilize green spaces during heat events. |

**Table 3: Environmental (Physical) Factors**

| **Factor** | **Impact on Heat Events and Green Space Benefits** |
| --- | --- |
| Shade Provision | Abundant shade can make green spaces more usable by offering protection from direct sunlight, while a lack thereof can lead to avoidance during peak heat. |
| Water Features | Cooling effects from water can enhance the thermal comfort of green spaces, lack of such features may reduce their appeal during hot weather. |
| Material and Design Choices | Reflective and heat-repelling materials in green space design can lessen heat absorption, whereas heat-absorbing surfaces can exacerbate local temperatures. |
| Green Infrastructure and Connectivity | An integrated network of green spaces and tree-lined routes promotes cooler environments and ease of access, while disjointed green assets can limit their usage. |
| Urban Planning and Land Use | Inclusive planning ensuring access to a variety of health behavior resources and cooling facilities promotes the utilization of green spaces, whereas inadequate planning leads to limited options during heat events. |

These tables succinctly convey how personal, social, and environmental factors can impact the use and benefits of green spaces during heat events. These factors should be considered collectively when planning and designing to ensure that such spaces remain accessible, safe, and beneficial regardless of temperature extremes.

1. The message that ChatGPT consistently remembers within a chat, see https://openai.com/blog/custom-instructions-for-chatgpt. [↑](#footnote-ref-1)
