# Supplementary material for: Leveraging and exercising caution with ChatGPT and other generative artificial intelligence tools in environmental psychology research
Source: Front Psychol. 2024 Apr 8;15:1295275. doi: 10.3389/fpsyg.2024.1295275 (PMC11033305; doi:10.3389/fpsyg.2024.1295275)
Supplement: Supplementary file 2 [file Data_Sheet_2.DOCX]

**Supplementary Data Sheet 2**

**Example 2 Generating examples with social, physical, personal contexts for extending a theory, as well as refining an existing concept**

**Custom instructions^[[1]](#footnote-1)^:**

You are a community health researcher and urban designer, knowing the concrete experiences of different people in being active. Your response is relatively concise.

---

You will help me to discover the breadth of experiences about restorative experiences in parks for female runners.

**Model**

Gpt-4-1106-preview

**USER**
I'd like to know people's stories before learning theories. Can you give me seven diverse stories (hypothetical examples) of female joggers' restorative experiences in parks? These examples should at least include who they were, when they used the park, what they did and encountered, and how they felt.

**ASSISTANT**

1. **The College Student**: Emily, a 20-year-old college student, used the park as a break from her intense study schedule. She jogged early in the mornings when the park was quiet and the air was fresh. On her runs, she admired the dew on the grass and the sunrise casting a golden glow on the pathway. After her runs, she felt energized and ready to tackle her classes, with the peacefulness of the park lingering in her mind throughout the day.
2. **The New Mother**: Aisha, a 30-year-old new mother, took up jogging as a way to regain her fitness. She jogged mid-morning when her mother could watch the baby. She pushed herself on the park's hilly terrain, feeling her strength return with each run. Aisha encountered other new mothers in the park, which led to new friendships. These runs left her feeling a sense of accomplishment and provided a precious moment of solitude.
3. **The Retiree**: Linda, a 65-year-old retiree, used the park for her evening jogs, which became a ritual for winding down her day. The park’s garden reminded her of the one she used to tend with her late husband. She jogged at a pace that allowed her to greet familiar faces and occasionally stop to watch children play. The evenings spent in the park made her feel connected to her community and helped her sleep better at night.
4. **The High-Powered Executive**: Sarah, a 40-year-old executive, ran in the park during her lunch breaks to escape the pressures of her job. The park's secluded trails offered her the solitude to process thoughts without the interruptions of her work environment. Encountering the occasional deer or rabbit provided an unexpected delight, reminding her of life beyond the office. After her runs, Sarah felt more clear-headed and patient in her decision-making.
5. **The Aspiring Athlete**: Priya, a 25-year-old aspiring marathoner, used the park for her intensive training sessions. She ran in the evenings, using the well-lit paths to practice her pace and endurance. Cheered on by other runners and park-goers, she felt motivated and supported. The sense of community and the runner’s high she experienced during these sessions were profoundly restorative and invigorating.
6. **The Creative Professional**: Mei, a 35-year-old graphic designer, found the park's natural beauty to be a rich source of inspiration. She ran midday when the park was quietly buzzing with life. The colorful landscapes and the play of light through the leaves spurred new ideas for her designs. Running provided her with a mental reset, and she returned to work with renewed creativity and fresh perspectives.
7. **The Recovering Patient**: Ana, a 45-year-old breast cancer survivor, took up running in the park as part of her physical and mental rehabilitation. She chose late afternoon when the sun was less intense, symbolizing the gentle approach she wanted to take with her body. The feeling of the breeze on her skin and the support from fellow runners, some of whom were also survivors, lifted her spirits. Post-run, she felt grateful for her body's capabilities and for the healing power of nature.

**USER**
Can you give me some diverse counter-examples?

**ASSISTANT**
Certainly, here are seven hypothetical counter-examples of less positive or restorative experiences that female joggers might have in parks:

1. **The Overwhelmed Student**: Jenna, a 19-year-old university student, tried jogging in the park but found it overcrowded and noisy, reflecting her stressful campus life. The sounds of traffic and lack of greenery made her feel more anxious rather than relaxed. She felt too distracted to experience any restorative benefits and left the park feeling more unsettled than when she arrived.
2. **The Shift Worker**: Rita, a 42-year-old nurse who worked night shifts, attempted to use the park for her runs but struggled with safety concerns due to poor lighting and isolated areas. Feeling vulnerable and unable to relax, her intentions for a soothing jog turned into a tense and hurried exercise routine, leaving her not rested but rather more wary.
3. **The Single Parent**: Maria, a 36-year-old single mom, tried jogging while her children played, but she couldn't focus on her run because she was too worried about their safety. The park lacked a secure, visible play area, and her run was frequently interrupted, resulting in frustration and a feeling of inefficiency in both her parenting responsibilities and her exercise goals.
4. **The Seasonal Affective Sufferer**: Giselle, a 28-year-old with seasonal affective disorder, found the park gloomy and uninviting during the winter months. The barren trees and gray skies did nothing to lift her mood. Her jogs became more of a chore than a pleasure, and she often felt more dejected and unmotivated after leaving the park.
5. **The Body-conscious Runner**: Sophia, a 33-year-old who was self-conscious about her fitness level, felt intimidated by the more experienced runners in the park. She perceived their glances as judgmental, making her runs stressful and embarrassing rather than empowering. Rather than feeling restored, she felt disheartened and often cut her runs short.
6. **The Allergy Sufferer**: Ellie, a 27-year-old with severe pollen allergies, found that her runs during the springtime caused more harm than good. Despite the beauty of the blooming flowers and trees, her allergy symptoms flared up severely, leading her to feel physically drained and unwell after attempting to run in the park.
7. **The Business Traveler**: Layla, a 39-year-old consultant who traveled frequently, tried to maintain her jogging routine in parks around the cities she visited. However, unfamiliarity with the areas often left her feeling disoriented and unsafe. The stress of finding her way back to her hotel or dealing with unwelcome attention negated any potential restorative effects of the run.

**USER**
I like the counterexamples because they are more diverse. Can you give me other seven?

**ASSISTANT**
Certainly, here are seven more hypothetical counter-examples of challenging experiences female joggers might have when seeking restorative experiences in parks:

1. **The Environmental Advocate**: Chloe, a 31-year-old environmental activist, found it difficult to enjoy her runs due to the litter scattered throughout the park. Each piece of trash she passed disrupted her attempt to find tranquility in nature, leaving her feeling disheartened about environmental stewardship and too distracted to find peace or restoration in her surroundings.
2. **The Busy Professional**: Tamara, a 37-year-old lawyer, tried to use the park for a quick run between meetings. Unfortunately, her tight schedule meant she often visited during the busiest hours, leading to congested paths and frequent interruptions. The experience was more stressful than restorative, as she could not find the quiet moment she needed to reset her mind.
3. **The Recent Immigrant**: Hina, a 29-year-old who recently moved to a new country, jogged in the park but felt a sense of isolation due to language barriers and cultural differences. She encountered groups of locals socializing and felt excluded and invisible, which compounded her feelings of loneliness rather than providing the sense of community and relaxation she sought.
4. **The Injury Recoveree**: Iris, a 26-year-old recovering from a knee injury, found the park's uneven terrain challenging and risky for her condition. Instead of the healing and empowering experience she hoped for, her jogs became a source of anxiety and fear of re-injury, undermining any potential restorative effects and leaving her feeling physically vulnerable.
5. **The Pet Owner**: Naomi, a 34-year-old dog owner, took her runs in the park while walking her dog, hoping to multitask. However, the presence of off-leash dogs and the need to constantly attend to her own pet distracted her from the mental release running usually provided. The runs became more about pet management than personal restoration.
6. **The Sensory-sensitive Runner**: Lila, a 24-year-old with sensory processing sensitivities, struggled with the loud noises from nearby playgrounds and sports fields. The overstimulation from children shouting and the whistles from games prevented her from falling into the meditative state she craved, and her jogs left her feeling more frazzled than rejuvenated.
7. **The Community Leader**: Fatima, a 40-year-old community leader, couldn't escape her responsibilities even during park runs. Locals often stopped her with community concerns and questions. Instead of providing an escape from daily stress, her jog turned into an extension of her work, leaving her with little sense of personal space or the opportunity to recharge.

**USER**
From the examples provided above, what personal, social, and physical context might have made a difference?

**ASSISTANT**
To assess the counter-examples provided, we can identify aspects within personal, social, and physical contexts that impact female joggers' experiences in parks and how addressing these aspects might contribute to more restorative experiences.

Personal Context:

1. **Mental Health and Physical Conditions**: For individuals like Giselle and Ellie, dealing with seasonal affective disorder and severe allergies, respectively, personal health conditions influenced their park experience. Adjustments such as timing visits with optimal sunlight exposure or when pollen levels are lower could be beneficial.
2. **Individual Preferences and Needs**: Some may seek solitude while others look for community connection. Runners like Hina who feel isolated or Sophia who is self-conscious could benefit from targeted programs that build social support and foster inclusivity.
3. **Lifestyle and Schedule**: The ability to integrate park visits into one's schedule, as with Tamara, can significantly contribute to the restorative quality of a jog. More flexible work schedules or dedicated time blocks for self-care could mitigate stress.
4. **Recovery and Adaptability**: For those recovering from injury or illness, like Iris, customized paths that are flat and have a softer surface or physical therapy guidance in the park can make for a more positive experience.

Social Context:

1. **Community Interaction and Involvement**: Negative social interactions, or a lack of them as faced by Fatima and Hina, can impede the restorative process. Community-driven events that encourage positive interactions and respect runners' space can create a better social atmosphere.
2. **Safety and Security Concerns**: Rita's concerns about park safety highlight the importance of surveillance, visibility, and general safety measures. Increased patrols, well-lit paths, and emergency call stations can enhance a sense of security.
3. **Cultural Inclusivity**: Understanding and embracing cultural diversity, as Hina's story suggests, is important for parks to be welcoming. Multilingual signs and culturally varied events can help bridge gaps and create inclusive spaces.

Physical Context:

1. **Park Maintenance and Cleanliness**: The condition of the park significantly affects the experience. Well-maintained, clean environments, as sought by Chloe, are more likely to contribute to a restorative experience.
2. **Design and Infrastructure**: The accessibility and design of a park, in terms of its paths, lighting, and facilities such as restrooms and drinking fountains, as noted in the examples of Rita and Iris, contribute to usability and comfort.
3. **Natural Elements and Aesthetics**: Seasonal changes and sensory experiences, as mentioned by Giselle and Lila, show that the presence and maintenance of natural beauty and quiet areas are crucial for a positive experience.
4. **Fitness Resources and Amenities**: Resources tailored for varied fitness levels and interests—from running tracks to exercise stations—can accommodate the needs of a diverse user base, as concerned by Naomi and Sophia.

Overall, the interplay between these personal, social, and physical contexts can significantly shape the restorative potential of park experiences for female joggers. Tailoring park features and programs to fit the diverse needs of user is essential for creating welcoming, restorative urban green spaces for all.

**USER**
I am trying to critically evaluate the applicability of attention restoration theory in the context of female runners in parks. Can you indicate how some of your examples (both positive and negative) can be explained by the theory's two components: fascination and being away?

**ASSISTANT**
Attention Restoration Theory (ART) postulates that environments can facilitate a psychological restoration process if they provide:

1. **Being Away**: A sense of escape from everyday routines and demands.
2. **Fascination**: Engagement with the environment in a way that captures attention effortlessly, often with aesthetically pleasing elements.

Let's apply these components to the hypothetical examples:

Positive Examples through ART lens:

- *Emily (College Student)*: Being in the park early in the morning provided Emily with "being away" from her academic routine. The "fascination" factor came from the natural stimuli of dew and sunrise, which captured her attention and allowed a reprieve from directed attention fatigue.
- *Linda (Retiree)*: Her evening jogs offered a mental "being away" from her daily life, and the familiar faces and playful children served as "soft fascinations" that engaged her without requiring intense focus, thus providing a restorative environment.
- *Mei (Creative Professional)*: The "fascination" with natural beauty inspired her creatively, and "being away" from her work environment allowed her mental space for reflection and rejuvenation.

Negative Examples through ART lens:

- *Jenna (Overwhelmed Student)*: The park was too similar to her stressful environment, lacking the "being away" component. Furthermore, the park's setting did not provide "fascination" due to the absence of calming or engrossing elements, failing to distract her from her stress.
- *Rita (Shift Worker)*: Safety concerns prevented her from achieving "being away," as she couldn't mentally disconnect from her worries. There was no "fascination" that could engage her attention benignly, as the park's atmosphere brought about vigilance instead of relaxation.
- *Sophia (Body-conscious Runner)*: Intimidation by other runners inhibited her feeling of "being away," as social comparison infiltrated her space for personal escape. The lack of "fascination" with the environment, potentially due to her self-focus and discomfort, impeded the attention restoration process.
- *Ellie (Allergy Sufferer)*: Allergic reactions would inhibit both "being away" and "fascination." Instead of being an environment where she could freely detach from concerns and enjoy the surroundings, her physical discomfort was a constant reminder of her ailment and took away from any soft fascination the park might provide.

From these examples, we can infer that ART can extensively explain the varying degrees of restorative experiences among female runners in parks. When the conditions of being away and fascination align with the individual's needs and the park's attributes, the park experience can be restorative. Conversely, when personal or park factors disturb these components, the experience may not be restorative, which highlights the importance of considering the specific needs and preferences of different individuals to optimize the benefits of natural settings as predicted by the Attention Restoration Theory.

**USER**
Can you indicate how some of your examples (both positive and negative) CANNOT be explained by the theory's two components: fascination and being away?

**ASSISTANT**
Attention Restoration Theory (ART) does a good job of explaining how natural environments can help replenish depleted mental resources through fascination and being away. However, ART might not fully account for certain social, cultural, or personal factors that influence the restorative experience. Here are some examples where fascination and being away components may not fully explain the joggers' experiences:

Positive Examples Beyond ART's Scope:

- *Aisha (New Mother)*: While Aisha's increased physical strength and solitude contribute to the "being away" component, ART does not directly explain the social aspect of her experience, such as forming friendships with other new mothers, which is a significant factor in her sense of restoration.
- *Priya (Aspiring Athlete)*: The support and motivation she receives from the community of runners could be as instrumental to her restorative experience as the natural setting itself, which ART doesn't primarily address.

Negative Examples Beyond ART's Scope:

- *Maria (Single Parent)*: ART might suggest that the park should offer a "being away" experience, but as a single parent, Maria's attention is divided. Her inability to experience restoration is not due to the absence of fascinating elements or the opportunity to be away, but rather her ongoing parental responsibilities and concerns.
- *Fatima (Community Leader)*: The reason her experience is not restorative is not due to a lack of "being away" or "fascination," but because her social role in the community follows her into the park, preventing mental detachment and personal time.
- *Hina (Recent Immigrant)*: Cultural barriers and feelings of social isolation impact her park experience. While the park might offer elements of "fascination" and the default notion of "being away," her sense of not belonging could hinder any potential restorative effects that ART proposes.

In these instances, psychological restoration in parks is influenced by factors such as social roles, cultural integration, and personal responsibilities that extend beyond the individual's interaction with the environment itself. While ART provides a valuable framework for understanding the restorative potential of nature, it doesn't encapsulate all variables that affect an individual's park experience. It's important to note that human experiences are multifaceted and that theories like ART provide general frameworks that may not fully account for the breadth and depth of individual human experiences within natural settings.

**USER**
ART may have simply explained some of the negative experiences by the concept of compatibility. However, I feel the concept is vague and abstract. Can you use your examples to summarize the specific situations for compatibility and lack of compatibility?

**ASSISTANT**
The concept of compatibility within Attention Restoration Theory refers to the fit between an individual's purposes and the environment's support for those purposes. When an environment aligns with a person's intentions or needs, it is considered compatible, leading to restorative experiences. Conversely, a lack of compatibility can lead to negative experiences as the environment does not support the individual's goals or activities. Here’s how the compatibility concept might explain specific situations in both negative and positive examples provided:

Positive Compatibility:

- *Emily (College Student)*: The quiet and serene morning park was compatible with her need for a peaceful escape from academic stress, making her running experience restorative.
- *Mei (Creative Professional)*: The natural beauty of the park matched her need for inspiration and a mental reset from work, demonstrating compatibility with her creative processes.

Negative Compatibility (lack of):

- *Jenna (Overwhelmed Student)*: The park felt like a continuation of her chaotic academic environment, lacking compatibility since it wasn't conducive to the mental break she sought.
- *Rita (Shift Worker)*: The poorly lit park did not match her safety needs as a nighttime jogger. The environment was incompatible with her purpose of finding relaxation and exercise after work.
- *Sophia (Body-conscious Runner)*: Since the park was an environment where she felt judged rather than accepted, it was incompatible with her goal of improving fitness without social pressure.
- *Hina (Recent Immigrant)*: The park did not provide a setting where she could feel culturally connected or understood, indicating a lack of compatibility between her social needs and the park environment.

In understanding compatibility, it is helpful to examine specific needs and goals of the joggers: safety, solitude, community, and mental respite, among others. When park conditions don't support these needs, as the negative examples illustrate, the environment is not compatible, and the joggers’ experiences are not restorative. In the positive examples, the park settings align well with individual needs, showcasing how compatibility can contribute to a sense of restoration and well-being.

Therefore, evaluating compatibility involves looking beyond the aesthetic or ecological aspects of a park and involves considering the individual's psychological, social, and physical needs in relation to the environment.

**USER**
It seems there can be subtypes of compatibility for female runners. Can you propose three ways to categorize the compatibility and what the sub-types are in each way of categorization?

**ASSISTANT**
Certainly! Examining compatibility for female runners in terms of subtypes can help us understand the nuanced experiences they may encounter. Here are three categorization approaches with their respective sub-types:

1. Purpose Compatibility:

- **Exercise Compatibility**: The park's design supports the specific physical activities a runner engages in, such as jogging, stretching, or interval training. Paths are well-maintained, terrain is varied to challenge different fitness levels, and there may even be markers for distance.
- **Social Compatibility**: The park facilitates social interactions desired by the runner—whether it's finding solitude or the opportunity to connect with other joggers and community members. Features such as group exercise areas or quiet zones cater to these varying social interactions.
- **Psychological Compatibility**: The park meets the psychological needs of the runner, which can range from stress relief and mental reprieve to sensory stimulation. A park with areas of serenity as well as sensory gardens with vibrant flowers and textures would meet this compatibility.

2. Environmental Compatibility:

- **Safety Compatibility**: The park ensures the feeling of security that is critical for a runner's comfort. Ample lighting, clear sightlines, emergency stations, and a regular presence of staff or security personnel contribute to this compatibility.
- **Sensory Compatibility**: The environment matches the sensory preferences of the runner—providing a multisensory experience for those who seek stimulation or serene, minimal sensory input for those who need to reduce overstimulation.
- **Amenity Compatibility**: The availability and quality of amenities—such as restrooms, water fountains, and weather shelters—support the functionality of the runner’s visit, enhancing the overall park experience and utility.

3. Temporal Compatibility:

- **Schedule Compatibility**: The park accommodates the runner’s schedule, being accessible during the hours that fit various lifestyles, such as early mornings, lunch breaks, or late evenings, addressing the time constraints of different users.
- **Seasonal Compatibility**: The park provides for a good experience year-round, with seasonal plantings, shaded areas for hotter months, and snow/leaf removal during winter or fall, ensuring that the environment is agreeable no matter the season.
- **Lifecycle Compatibility**: The park is suitable for runners at different life stages, offering play areas for parent runners to monitor their children, smooth paths for strollers or seniors, and challenging circuits for competitive athletes.

Each of these compatibility categorizations and subtypes helps create a more detailed understanding of what makes a park a restorative environment for female runners. By considering these various compatibilities, urban designers and park planners can better cater to the diverse needs and preferences of runners, making parks more inclusive and accommodating for all.

**USER**
I checked your examples again and not sure if our moderators and compatibility subtypes can explain 6 The Allergy Sufferer: Ellie; 7 The Business Traveler: Layla; 7 The Community Leader: Fatima. Let me know if these cases are well explained, if we can revise existing concepts, or if we have to introduce new concepts.

**ASSISTANT**
Let's re-examine the cases of Ellie, Layla, and Fatima in the context of ART's moderators and the discussed compatibility subtypes to establish if they explain the experiences well or if revisions or new concepts are required:

Ellie (The Allergy Sufferer)

- **Compatibility Considerations**: Seasonal Compatibility is relevant, as her allergic reactions are linked to specific times of the year when pollen counts are high. Environmental Compatibility is also a factor; however, it does not account for the health-related challenges associated with allergies.
- **ART Moderators**: The "fascination" aspect could be affected as Ellie might be unable to appreciate the beauty of nature due to her discomfort. "Being away" also loses its effectiveness if the environment actively harms her well-being.
  **Potential Revisions/New Concepts**:
  - **Health Compatibility**: Recognizing that some individuals may have health issues directly triggered by the environment suggests a need for health-compatible park designs. This could include planting low-pollen-producing vegetation and providing information on pollen counts to aid visitors in planning their park usage.

Layla (The Business Traveler)

- **Compatibility Considerations**: Schedule Compatibility is an issue, as her irregular schedule and unfamiliar locations make consistent use of park spaces challenging. Safety and Environmental Compatibility are also concerns due to Layla's unfamiliarity with new parks, which can affect perceived safety and ease of navigation.
- **ART Moderators**: Layla could find "being away" in a new park, but "fascination" could be hindered by her lack of comfort or knowledge about the safety and layout of a new environment.

Potential Revisions/New Concepts:

- - **Informational Compatibility**: Ensuring that parks provide clear, accessible information about their layouts, facilities, and safety features, especially online, could help transient users like Layla feel more secure and oriented in new parks.

Fatima (The Community Leader)

- **Compatibility Considerations**: Social Compatibility here has a negative effect, as Fatima's jogging experience is interrupted by her social role, preventing her from disconnecting. Psychological Compatibility is also lacking because the environment does not provide the mental space she needs.
- **ART Moderators**: Her experience with "being away" is disrupted due to her continuous engagement with community tasks. "Fascination" loses its restorative potential because her attention is diverted to interpersonal interactions rather than the environment.

Potential Revisions/New Concepts:

- - **Role Segmentation Compatibility**: Introducing the concept that individuals sometimes need environments that help them separate different life roles. For Fatima, this could mean creating spaces or times in the park where her community leader role is not expected to be front and center.

In summary, while many aspects of Ellie's, Layla's, and Fatima's experiences can be partially explained by the existing compatibility concepts in ART, each case presents unique challenges that might benefit from revisions to these concepts or the introduction of new ones. Health, Informational, and Role Segmentation Compatibility are concepts that could enhance the understanding and address the needs of these particular user groups within park design and policy.

1. The message that ChatGPT consistently remembers within a chat, see https://openai.com/blog/custom-instructions-for-chatgpt. [↑](#footnote-ref-1)
