# Supplementary material for: Leveraging and exercising caution with ChatGPT and other generative artificial intelligence tools in environmental psychology research
Source: Front Psychol. 2024 Apr 8;15:1295275. doi: 10.3389/fpsyg.2024.1295275 (PMC11033305; doi:10.3389/fpsyg.2024.1295275)
Supplement: Supplementary file 3 [file Data_Sheet_3.DOCX]

**Supplementary Data Sheet 3**

**Example 3**. **Creating a pool of factors and indicators for an environment audit tool for the stress relieving (restorativeness) potential of a university campus**

**Custom instructions^[[1]](#footnote-1)^:**

You are a health psychologist with experience in psychometrics. You will help me propose key constructs and indicators for an environment audit tool. You'd like to give an exhaustive list using concise language.

**Model**

Gpt-4-1106-preview

**USER**
We are developing an environmental audit tool for the stress relieving potential of the university campus environment. We need a list of possible high-impact factors.

**ASSISTANT**
When developing an environmental audit tool for assessing the stress-relieving potential of a university campus environment, you'll want to consider a range of factors that can impact students' psychological well-being. Here's a list of key constructs and their potential indicators:

1. Natural Elements:
   - Presence of green spaces (lawns, gardens)
   - Variety and health of plant life (trees, flowers, shrubs)
   - Accessibility to natural areas (walking paths, seating in green areas)
   - Water features (fountains, ponds)
2. Built Environment:
   - Building design and aesthetics (architecture, color schemes)
   - Maintenance and cleanliness of facilities
   - Availability of quiet study spaces
   - Adequacy of lighting, both natural and artificial
   - Ergonomics of furniture and spaces
3. Ambient Conditions:
   - Noise levels in and around campus
   - Air quality (indoor and outdoor)
   - Temperature control and comfort
   - Presence of restorative spaces (areas designed for relaxation and rejuvenation)
4. Safety and Security:
   - Visibility of security personnel
   - Adequacy of security measures (lighting, emergency phones)
   - Students' perceptions of safety on campus
5. Social Spaces:
   - Availability and quality of communal areas (lounges, cafeterias)
   - Opportunities for social interaction and community building
   - Inclusivity of spaces for diverse student populations
6. Recreational Facilities:
   - Access to sports and fitness facilities
   - Availability of recreational programs and clubs
   - Quality and maintenance of equipment
7. Wayfinding and Signage:
   - Clarity and visibility of signage
   - Ease of navigation throughout the campus
   - Availability of maps and directional information
8. Aesthetic and Sensory Experience:
   - Visual appeal of the campus environment
   - Presence of art or other aesthetic installations
   - Sensory stimulation (visual, auditory, olfactory)
9. Connectivity to Surrounding Environment:
   - Ease of access to off-campus amenities (shops, parks)
   - Quality of transition spaces between campus and community
   - Transportation options (bike lanes, public transit access)
10. Sustainability Practices:
    - Visibility of recycling and waste reduction efforts
    - Energy efficiency measures in place
    - Use of sustainable and eco-friendly materials
11. Technological Resources:
    - Availability and reliability of Wi-Fi connectivity
    - Access to technological support and resources
    - Integration of technology into the learning environment
12. Cultural and Spiritual Elements:
    - Presence of cultural centers or resources
    - Availability of spiritual or meditative spaces
    - Representation of campus cultural diversity

Each of these constructs can be measured using a variety of methods, including observational checklists, surveys of student perceptions, objective measures (e.g., decibel levels for noise), and usage statistics (e.g., frequency of use of green spaces or fitness centers). The tool should be designed to capture both the objective features of the environment and the subjective experiences of the individuals within it, as both contribute to the stress-relieving potential of the campus.

**USER**
What can be some categories of those factors? Give me three ways to categorize them

**ASSISTANT**
The factors listed above can be categorized in several ways depending on the focus of the environmental audit. Three possible ways to categorize these factors are:

1. Physical vs. Social Environment:
   - **Physical Environment:** This includes all tangible aspects such as natural elements, built environment, ambient conditions, recreational facilities, wayfinding and signage, sustainability practices, and technological resources.
   - **Social Environment:** This encompasses the social aspects of the environment, including safety and security, social spaces, opportunities for social interaction, cultural and spiritual elements, and the overall atmosphere that influences social relationships and a sense of community.
2. Objective vs. Subjective Factors:
   - **Objective Factors:** These are measurable features of the environment that can be quantified or assessed through observation, such as noise levels, air quality, maintenance of facilities, and availability of spaces and resources.
   - **Subjective Factors:** These refer to individuals' perceptions and experiences of the environment, such as feelings of safety, aesthetic appeal, and the perceived adequacy of communal areas and recreational programs.
3. Static vs. Dynamic Factors:
   - **Static Factors:** These are relatively stable features of the environment that do not change frequently, such as the presence of green spaces, building architecture, and the layout of the campus.
   - **Dynamic Factors:** These include elements that can change more rapidly, either by design or through use, such as the cleanliness of facilities, the organization of social events, and the implementation of new sustainability practices or technological upgrades.

By categorizing the factors in these ways, the audit tool can be structured to systematically assess different dimensions of the university campus environment and its potential to alleviate stress among students. This categorization also helps in tailoring interventions and improvements to specific aspects of the campus environment.

**USER**
I think the social vs physical is better. Can you propose a list without the detailed subpoints

**ASSISTANT**
Certainly, here's a streamlined list of factors categorized under Physical and Social environments:

Physical Environment:

- Natural Elements
- Built Environment
- Ambient Conditions
- Recreational Facilities
- Wayfinding and Signage
- Sustainability Practices
- Technological Resources

Social Environment:

- Safety and Security
- Social Spaces
- Cultural and Spiritual Elements
- Community Building Opportunities
- Inclusivity of Spaces

**USER**
Can you rate the importance of the factors for stress relieving from 1-5. Also, explain why. The purpose is to drop some less relevant factors

**ASSISTANT**
Rating the importance of environmental factors for stress relief is a subjective exercise and can vary depending on the specific context of the university and its student population. However, I can provide a general rating based on the typical impact these factors may have on stress relief, along with a brief explanation for each:

Physical Environment:

1. Natural Elements - 5/5: Exposure to nature has been consistently linked to stress reduction, improved mood, and overall well-being.
2. Built Environment - 4/5: The design and maintenance of buildings can influence comfort levels and stress, particularly in learning environments.
3. Ambient Conditions - 4/5: Factors like noise, air quality, and temperature can significantly affect concentration, relaxation, and physiological stress responses.
4. Recreational Facilities - 3/5: While important for physical health and leisure, the direct impact on stress relief can be more individualized.
5. Wayfinding and Signage - 2/5: Clear navigation can reduce frustration and anxiety, but it may have a less direct impact on overall stress levels.
6. Sustainability Practices - 2/5: Although important for long-term environmental health, the immediate impact on individual stress relief might be less noticeable.
7. Technological Resources - 3/5: Reliable technology can reduce academic stress, but it may not directly contribute to relaxation or stress relief.

Social Environment:

1. Safety and Security - 5/5: Feeling safe is fundamental to stress reduction and is a primary concern for well-being on campus.
2. Social Spaces - 4/5: Opportunities for socializing and support can be crucial for emotional stress relief and a sense of belonging.
3. Cultural and Spiritual Elements - 3/5: These factors contribute to personal identity and community, which can be stress-relieving for some students.
4. Community Building Opportunities - 4/5: Engagement in community and social networks can provide support systems that alleviate stress.
5. Inclusivity of Spaces - 4/5: Inclusive environments can reduce social stress and promote a sense of safety and belonging for all students.

In deciding which factors to prioritize, it's important to consider the unique needs and characteristics of your student population. For example, if your campus is in a densely urban area with little green space, enhancing natural elements might be particularly impactful. Similarly, if there are concerns about campus safety, prioritizing safety and security measures would be crucial.

The ratings above assume a general campus environment, but actual priorities should be informed by a thorough needs assessment that includes student input, campus-specific challenges, and current research on environmental psychology.

**USER**
Great, we will only keep the factors rated as 3 or above. Also, if a group of auditors needs to audit a campus, what are some key qualities should indicators have to make their audit more reliable and easier? Give me the five most important qualities.

**ASSISTANT**
To ensure that an audit is reliable and efficient, the indicators used should possess the following key qualities:

1. **Measurability:**
   Indicators should be quantifiable or observable to allow for objective measurement. This ensures that different auditors can assess the same factor and obtain similar results, enhancing inter-rater reliability.
2. **Specificity:**
   Each indicator should be clearly defined and specific to the factor it is intended to measure. This reduces ambiguity and ensures that all auditors understand exactly what to look for during the assessment process.
3. **Relevance:**
   Indicators must be directly related to the constructs they are intended to measure and should have a demonstrated connection to stress relief. This ensures that the audit focuses on factors that genuinely impact the stress-relieving potential of the environment.
4. **Sensitivity:**
   Indicators should be sensitive enough to detect even small changes in the environment. This allows auditors to identify areas of improvement and to monitor changes over time.
5. **Actionability:**
   The indicators should provide information that can lead to actionable recommendations. This means that they should not only identify areas of concern but also suggest potential interventions that could be implemented to enhance the stress-relieving aspects of the campus environment.

By incorporating indicators with these qualities into the audit tool, auditors will be better equipped to conduct assessments that are consistent, reliable, and useful for making informed decisions to improve the campus environment for stress relief.

**USER**
Now propose 5-10 best indicators you can think of for the first factor. The specific number should depend on the availability of good indicators.

**ASSISTANT**
Given the importance of natural elements in stress reduction, here are seven indicators that could effectively measure this factor in a university campus environment:

1. Quantity of Green Spaces:
   - Indicator: The total area of green spaces (gardens, lawns, courtyards) as a percentage of the campus footprint.
2. Quality of Green Spaces:
   - Indicator: The health and maintenance level of plant life within these spaces, assessed through a standardized checklist.
3. Accessibility of Green Spaces:
   - Indicator: The number of accessible green spaces within a 5-minute walk from major campus buildings.
4. Diversity of Plant Life:
   - Indicator: The variety of plant species present, which contributes to biodiversity and the aesthetic quality of green spaces.
5. Usage of Green Spaces:
   - Indicator: Frequency of use of green spaces by students and staff, determined by observational counts or surveys.
6. Presence of Seating and Amenities in Green Spaces:
   - Indicator: The number and quality of benches, tables, and other amenities that encourage relaxation and social interaction within green areas.
7. Water Features:
   - Indicator: The presence and maintenance of water features (e.g., fountains, ponds) which can enhance the calming effect of natural spaces.

These indicators provide a comprehensive assessment of the natural elements on a university campus, capturing both the physical presence and the quality of green spaces that contribute to stress relief. They are measurable, specific, and actionable, which will aid auditors in evaluating and recommending improvements to the campus environment.

**USER**
The next physical environment factor

**ASSISTANT**
The next factor to consider is the "Built Environment," which encompasses the design, infrastructure, and maintenance of campus buildings and facilities. Here are six indicators that can effectively measure this factor:

1. Architectural Aesthetics:
   - Indicator: Rating of the visual appeal and design harmony of campus buildings, using a standardized aesthetic assessment tool.
2. Building Maintenance:
   - Indicator: Level of upkeep and repair of campus buildings, assessed through a maintenance checklist or facility condition index.
3. Availability of Quiet Study Areas:
   - Indicator: Number and capacity of designated quiet study spaces relative to student population.
4. Lighting Quality:
   - Indicator: Adequacy and comfort of lighting in campus buildings, including a mix of natural and artificial light sources, evaluated through light level measurements and user satisfaction surveys.
5. Ergonomics and Comfort:
   - Indicator: Assessment of furniture and space ergonomics in classrooms, libraries, and study areas, using ergonomic guidelines and student feedback.
6. Indoor Air Quality:
   - Indicator: Measurement of indoor air pollutants and ventilation effectiveness, using air quality monitoring devices.

These indicators are designed to capture the key aspects of the built environment that can influence stress levels among university students and staff. They are selected for their direct impact on daily experiences on campus and their potential to improve the overall well-being of the campus community.

**USER**
The next physical environment factor

**ASSISTANT**
The next physical environment factor to consider is "Ambient Conditions," which refers to the sensory aspects of the environment that can influence stress levels. Here are five indicators that can be used to assess ambient conditions:

1. Noise Levels:
   - Indicator: Decibel readings at various locations on campus during different times of the day to assess compliance with acceptable noise standards.
2. Air Quality:
   - Indicator: Concentrations of common air pollutants (e.g., PM2.5, CO2 levels) measured at various indoor and outdoor locations on campus.
3. Thermal Comfort:
   - Indicator: Temperature and humidity readings in conjunction with student and staff comfort surveys to evaluate the adequacy of heating, ventilation, and air conditioning (HVAC) systems.
4. Lighting Conditions:
   - Indicator: Evaluation of the adequacy of both natural and artificial lighting in terms of intensity and comfort, using light level measurements and qualitative assessments.
5. Olfactory Environment:
   - Indicator: Incidence of reported unpleasant odors or assessments of olfactory conditions in different campus areas, noting potential sources of odor pollution.

These indicators are critical for assessing the sensory environment that can impact stress and comfort levels for individuals on campus. They are measurable and actionable, allowing for targeted interventions to improve the overall ambient conditions of the university environment.

**USER**
The next physical environment factor

**ASSISTANT**
The next physical environment factor to consider is "Recreational Facilities," which includes the spaces and resources provided for physical activity, leisure, and relaxation. Here are five indicators that can be used to assess recreational facilities:

1. Availability of Recreational Spaces:
   - Indicator: The number of recreational facilities (e.g., gyms, sports fields, swimming pools) relative to the student population.
2. Condition and Maintenance of Facilities:
   - Indicator: The current state of repair and maintenance of recreational facilities, assessed through regular inspections or maintenance logs.
3. Access to Recreational Programs:
   - Indicator: The variety and frequency of recreational programs and activities offered, including intramural sports, fitness classes, and wellness workshops.
4. Equipment Quality and Variety:
   - Indicator: The availability and condition of equipment in fitness centers and sports facilities, evaluated through inventory checks and user feedback.
5. Utilization of Recreational Facilities:
   - Indicator: Usage rates of recreational facilities, determined by tracking attendance or membership usage data.

These indicators help to assess the capacity of the campus to provide physical activity opportunities, which are known to be effective for stress management and overall well-being. They are designed to be measurable, providing clear data points for evaluating the adequacy and impact of recreational facilities on campus.

**USER**
Those indicators are hard to measure, considering the number of facilities and types of equipment can be large. And the conditions can be subjective to the specific auditors. What could be two other strategies to create the indicators?

**ASSISTANT**
When creating indicators for recreational facilities that are more manageable and less subjective, consider the following two strategies:

1. Simplified Checklists and Ratings:
   - Develop a standardized checklist that captures the essential elements of recreational facilities, such as cleanliness, safety, and general condition. This checklist should be simple enough for auditors to use quickly but comprehensive enough to cover key aspects of facility quality.
   - Implement a rating system for the condition of facilities and equipment. For example, a scale from 1 (poor) to 5 (excellent) can standardize how auditors assess the quality, with clear criteria for what each rating entails.
2. User Satisfaction Surveys:
   - Distribute surveys to students and staff to gather subjective assessments of the recreational facilities. This can include questions about satisfaction with the availability, condition, and variety of facilities and programs.
   - Collect data on perceived barriers to using the facilities, such as inconvenient hours, overcrowding, or lack of desired equipment or programs.

By using these strategies, you can create indicators that are both easier to measure and less dependent on individual auditor judgment. The combination of objective checklists with subjective

User feedback provides a more rounded and reliable assessment of the recreational facilities' contribution to stress relief on campus.

**USER**
Can you create an example scale for the facility condition to make it easier to measure?

**ASSISTANT**
Certainly! Below is an example of a 5-point Likert scale designed to assess the condition of recreational facilities. This scale can be used by auditors to rate various aspects of the facilities, such as equipment, spaces, and overall maintenance.

Recreational Facility Condition Scale:

1. Poor (1):
   - Facilities are in disrepair or unsafe for use.
   - Equipment is outdated, damaged, or frequently out of order.
   - The space is unclean and not well-maintained.
   - Immediate action is required to address safety and usability concerns.
2. Fair (2):
   - Facilities show signs of wear and require attention.
   - Some equipment is old or occasionally malfunctioning.
   - The space is somewhat clean, but maintenance is inconsistent.
   - Improvements are needed to meet basic standards for comfort and safety.
3. Good (3):
   - Facilities are functional but may not be state-of-the-art.
   - Equipment is generally in working order, with some items showing moderate use.
   - The space is clean, with regular maintenance apparent.
   - Some updates are recommended to enhance
   - User experience.
4. Very Good (4):
   - Facilities are well-maintained with minor signs of use.
   - Equipment is modern and well-maintained, with rare malfunctions.
   - The space is consistently clean and inviting.
   - Minor enhancements could be made, but overall, the facilities meet
   - User needs effectively.
5. Excellent (5):
   - Facilities are in excellent condition with top-of-the-line features.
   - Equipment is new or like-new, with a high standard of maintenance.
   - The space is spotless, with a clear commitment to high-quality maintenance.
   - The facilities exceed expectations and provide an outstanding
   - User experience.

This scale can be used to rate individual aspects of the recreational facilities or as an overall assessment. It provides clear criteria for each level, reducing subjectivity and ensuring consistency across different auditors. The ratings can be averaged to give an overall score for the facility or used to identify specific areas in need of improvement.

1. The message that ChatGPT consistently remembers within a chat, see https://openai.com/blog/custom-instructions-for-chatgpt. [↑](#footnote-ref-1)
