# Supplementary material for: Leveraging and exercising caution with ChatGPT and other generative artificial intelligence tools in environmental psychology research
Source: Front Psychol. 2024 Apr 8;15:1295275. doi: 10.3389/fpsyg.2024.1295275 (PMC11033305; doi:10.3389/fpsyg.2024.1295275)
Supplement: Supplementary file 4 [file Data_Sheet_4.DOCX]

**Supplementary Data Sheet 4**

**Example 5. Transferring a description of the procedure from a citation into a training manual, an IRB application, and a consent form**

**Custom instructions^[[1]](#footnote-1)^:**

You are an experienced experimental psychologist. We are going to create documents for a research project. Your answer needs to be tailored to the specific type of document.

**Model**

Gpt-4-1106-preview

**USER**

Please briefly summarize the key phases of this methods section from an existing paper^[[2]](#footnote-2)^: "some scenes will appear in VR headset, you should look freely as you do in daily life. You can also walk around in a small circle. If you go out of the accessible area, the experimenter will alert you”. First, the participant was immersed in a test scene adapt to the VR environment for 1 min. Then, the scene changed to the experimental scene (Figs. 1–2), in which the participant could view freely for another 1 min, without any other prompts. During the entire VR process, eye-tracking data were recorded implicitly. The whole time spent by one participant was 10–15 min. The procedure was designed based on the following concerns. First, we adopted free viewing to predict visual attention like other researchers (Berto, Massaccesi & Pasini, 2008; Parkhurst, Law, & Niebur, 2002). Second, the direction of the starting gaze was random, so each side of the 360-degree image had an equal probability of being seen first. Third, the stimulus exposure time came from a pilot test.1 One minute was enough for a participant to fully explore the whole scene without feeling bored and turning to fix gaze on a point. One week after the experiment, all participants’ NR score were collected through an online questionnaire. The one-week interval was designed to eliminate any potential interference between the VR environmental stimulus and the questionnaire survey."

**ASSISTANT**
The methods section outlines the procedures used to immerse participants in a virtual reality (VR) environment to investigate their visual attention. The key phases can be summarized as follows:

1. Acclimatization Phase:
   - Participants were first introduced to the VR setting with a test scene for one minute to get accustomed to the environment.
2. Free Viewing and Recording:
   - The test scene then switched to the experimental scene, which participants could view freely for another minute.
   - Participants were allowed to walk in a small area, with the experimenter ensuring they stayed within the designated space.
   - Eye-tracking data were collected silently throughout the VR experience.
3. Duration of VR Experience:
   - Each participant spent between 10 to 15 minutes in the VR environment.
4. Design Considerations:
   - The decision to allow free viewing was to align with prior research on predictive visual attention.
   - The starting gaze direction was randomized to ensure all parts of the 360-degree scene had an equal chance of being observed first.
   - The one-minute exposure time for each scene was established through a pilot test and aimed to prevent boredom and maintain engagement.
5. Follow-Up:
   - A week after the VR experiment, participants completed an online questionnaire to provide their 'NR score'.

The procedure incorporated measures to produce a natural visual experience, avoid biases in initial attention, and gather data on participants' visual attention without external prompts or pressures.

**USER**
Please combine phases 2-5 into phase 2 - free viewing and recording. Now we are going to expand the description into detailed instruction for research assistants. Please expand the phase 1 acclimatization

**ASSISTANT**
Phase 1: Acclimatization to the VR Environment

As a research assistant, you will be responsible for guiding participants through the initial phase of acclimatization to the virtual reality (VR) environment. This phase is critical for ensuring participants are comfortable with the VR headset and the environment. Below are the detailed steps you'll need to follow:

1. Participant Preparation:
   - Greet the participant and explain that the study involves a VR experience.
   - Obtain informed consent and ensure the participant has no pre-existing conditions that might contra-indicate VR use (e.g., epilepsy, severe motion sickness).
2. Introduction to VR Headset:
   - Introduce the VR headset to the participant, explaining its function and how to wear it comfortably.
   - Adjust the straps and lenses to ensure a snug fit and clear vision for the participant.
3. Test Scene Introduction:
   - Once the headset is on, start with the test scene, which is designed to help the participant acclimate to the VR.
   - Instruct the participant to explore the test scene for one minute, emphasizing that they should look around as they would in everyday life.
   - Let them know they can turn their head to look at different parts of the scene or walk around a little if they wish.
4. Movement and Safety:
   - As participants may walk during the acclimatization phase, delimitate a small area (e.g., within a small, defined circle) where they can move safely.
   - Monitor the participant's movement to ensure they stay within the safe area. As they walk, ensure they do not bump into real-world obstacles.
   - Verbally guide the participant back to the safe zone if they reach the boundaries of the accessible area.
5. Acclimatization Completion:
   - After the one-minute acclimatization period, confirm whether the participant feels comfortable and ready to proceed.
   - Address any issues or discomforts reported by the participant before moving on to the main phase of the study.
6. Safety and Comfort Checks:
   - Throughout the acclimatization phase, observe the participant for signs of discomfort or distress.
   - Check in with the participant periodically to make sure they are not experiencing motion sickness or any other adverse effects.
7. Transition to Experimental Scene:
   - Once the participant is acclimated and confirms their readiness, explain that the scene will change to the experimental scene where the formal data collection process will begin.
   - Assure the participant that you will be there to assist them throughout the experience.

As the participant moves through this acclimatization phase, be ready to provide assistance and answer any questions they may have. Your role in ensuring the participant's comfort and safety is critical to the success of this experimental procedure.

**USER**
Please expand the phrase 2

**ASSISTANT**
Phase 2: Free Viewing and Recording Procedure

In this phase, you'll oversee the experimental procedure, where participants engage in free viewing of the VR scene while eye-tracking data is recorded. The following are detailed instructions for research assistants to ensure consistency and accuracy in data collection:

1. Experimental Scene Transition:
   - Inform the participant that the test scene will switch to the experimental scene and reinforce that they should continue to look around freely.
2. Eye-Tracking Calibration:
   - Prior to beginning the session, calibrate the eye-tracking system with the participant's gaze.
   - Ensure that the eye-tracker is properly recording data by running a brief pre-test, and make any necessary adjustments.
3. Free Viewing Instructions:
   - Advise the participant that they will have one minute to view the experimental scene freely without any guidance or specific tasks.
   - Remind them to explore the scene as they would naturally in real life and reiterate that they have the freedom to walk around within the designated safe zone.
4. Monitoring Movement:
   - Continuously observe the participant to ensure they stay within the safe area as they explore the VR environment.
   - Be prepared to gently alert the participant verbally if they are approaching the boundaries of the accessible area to prevent any accidents.
5. Data Recording:
   - Start the eye-tracking data recording as soon as the experimental scene commences.
   - Monitor the recording equipment to ensure it is functioning correctly and data are being recorded without issue.
6. Time Management:
   - Keep track of the one-minute viewing time using a stopwatch or timer.
   - After one minute has elapsed, prompt the participant to stop viewing and prepare for the next step in the procedure.
7. Post-Viewing Process:
   - Once the participant has completed the free viewing of the scene, confirm that the eye-tracking data collection is properly concluded and saved.
   - Conduct a brief verbal check-in with the participant to ensure they did not experience discomfort during the viewing.
8. Follow-Up Information:
   - Inform the participant about the one-week follow-up where they will be asked to complete an online questionnaire.
   - Explain the purpose of the follow-up and how it contributes to the study.
   - Collect participant contact information as appropriate for the follow-up process and provide instructions on how they will receive the questionnaire.
9. Debriefing:
   - Explain to the participant the overall objectives of the research study to provide context for the activity they've just completed.
   - Answer any questions they may have about the study or their participation.

By meticulously following these steps, you will help ensure a smooth and consistent experimental procedure that values participant safety and comfort while collecting high-quality data for analysis.

**USER**
Can you create the script for the first phase?

**ASSISTANT**
Certainly! Here is a script tailored for research assistants to guide participants through the first phase of acclimatization to the VR environment:

**Research Assistant Script for Acclimatization Phase**

**Introduction:**

"Hello and welcome! Thank you for participating in our study today. Before we begin, I need to confirm that you've read and signed the informed consent form. Great! Have you ever experienced virtual reality before, or do you have any conditions like epilepsy or severe motion sickness that we should be aware of?"

[Wait for the participant's response and address any concerns.]

**VR Headset Introduction:**

"This is the VR headset that we'll be using today. It's going to transport you into a virtual world where you'll be able to look around as if you were in a different place. I'll help you put it on and adjust it to make sure it's comfortable. Let's get started."

[Assist the participant with putting on the headset and make adjustments as necessary.]

**Acclimatization Instructions:**

"In just a moment, you'll see a test scene appear in the VR headset. This is just to help you get used to the environment. Please look around freely, just as you would in everyday life. You can also walk around within this small circle I've marked on the floor. If you step outside this area, I'll let you know so that you can step back inside. Are you ready to begin?"

[Wait for the participant's confirmation.]

**During Acclimatization:**

[Start the test scene and monitor the participant.]

"Please take this minute to look around and explore the scene. Feel free to turn your head to see different parts, and if you'd like, you can walk around a bit. Remember to stay within the marked area for your safety."

[Keep an eye on the participant's movement and provide guidance as needed.]

**Acclimatization Completion:**

"That's been one minute. How do you feel? Are you comfortable with the VR headset and the environment?"

[Address any issues or discomfort the participant may express.]

"If everything feels good, we're going to transition to the experimental scene where we will begin the main part of the study. Remember, there are no right or wrong ways to look around, and you can continue to move within the safe area. I'll be here throughout the process if you need any assistance."

[Ensure the participant is ready before moving on to the next phase.]

Feel free to adapt this script to the specific needs of your study and the comfort level of your participants. The goal is to ensure participants are comfortable and informed about the process before the actual data collection begins.

**USER**
What may be some unexpected issues/questions from participants and how can the research assistant respond? Please propose five likely issues/questions.

**ASSISTANT**

1. **Issue/Question**: "I'm feeling a bit dizzy/nauseous while using the VR headset. Is this normal?"

**Response**: "Some people do experience a sensation of dizziness or nausea when they first use VR, which is often called 'VR sickness.' It's similar to motion sickness. If you're feeling uncomfortable, we can take a break, and you can remove the headset. Your well-being is our top priority, so please let me know how you're feeling throughout the experiment."

1. **Issue/Question**: "The VR headset is blurry, and I can't see the scene clearly. Can it be adjusted?"

**Response**: "Absolutely, let's make sure the headset fits you properly. Sometimes the lenses need to be adjusted to match your eyesight better. We can also check if the headset's position needs to be shifted or if the straps need tightening. Tell me if the image becomes clearer as I make these adjustments."

1. **Issue/Question**: "What should I be looking for in the scene? Is there a specific task I need to complete?"

**Response**: "For this part of the study, there's no specific task for you to complete. We're interested in your natural viewing behavior, so please look around the scene as you would naturally. There's no need to search for anything in particular; just explore the environment as you feel inclined."

1. **Issue/Question**: "The VR environment feels a bit overwhelming. Can I take the headset off for a moment?"

**Response**: "Of course, your comfort is very important to us. If at any point you feel overwhelmed, you can remove the headset. Just let me know, and we can pause the experiment. We can resume whenever you feel ready, or if you prefer, we can stop your participation here. Your health and comfort come first."

1. **Issue/Question**: "I accidentally stepped out of the safe area. Did I mess up the experiment?"

**Response**: "Not at all, stepping out of the safe area doesn't affect the experiment's data. The safe area is just there to ensure your safety so that you don't bump into anything while you're immersed in the VR environment. I'll help guide you back into the safe zone, and we can continue from there. Just focus on experiencing the scene as you would naturally."

In each of these responses, the research assistant should maintain a calm and reassuring demeanor, addressing the participant's concerns with empathy and professionalism. The assistant should also be prepared to pause the experiment or provide additional instructions as needed to ensure the participant's well-being and the integrity of the study.

**USER**
Great! Now we are going to create some text for IRB. Please rewrite a brief description of the study procedure with the three main phases. Each phase should include 2-3 sentences. Use plain and concise language

**ASSISTANT**
Title: Investigation of Visual Attention in Virtual Reality Environments

Description of Study Procedure:

Phase 1: Acclimatization
Participants will first become familiar with the virtual reality (VR) equipment. They will wear a VR headset and view a non-experimental scene for one minute to adjust to the virtual environment, during which they may look around and walk within a small, marked area for safety.

Phase 2: Free Viewing and Data Collection
Following acclimatization, participants will experience the main experimental scene for one minute, where they are free to look around without specific instructions. Their eye movements will be tracked to study where they naturally focus their attention in the VR scene, and they will remain within the safe area to prevent any accidents.

Phase 3: Follow-Up
One week after the VR session, participants will complete an online questionnaire to measure their responses to the VR experience. This follow-up is designed to assess any longer-term effects of the VR environment on participants' visual attention and memory of the experience.

This description outlines the progression of the study from initial acclimatization to the VR environment, through the main data collection phase, and concludes with a follow-up to capture post-experimental information.

**USER**
Now moving to consent form. Please summarize the procedure into three sentences, in plain language

**ASSISTANT**
In this study, you will wear a virtual reality headset to view and interact with different scenes, just as you might look around in real life. While you explore these scenes freely, we will be recording where you look using eye-tracking technology within the headset. After the VR experience, we'll ask you to complete a short online survey about your experience one week later.

1. The message that ChatGPT consistently remembers within a chat, see https://openai.com/blog/custom-instructions-for-chatgpt. [↑](#footnote-ref-1)
2. Chen, B., Gong, C., & Li, S. (2022). Looking at buildings or trees? Association of human nature relatedness with eye movements in outdoor space. Journal of Environmental Psychology, 80, 101756. https://doi.org/10.1016/j.jenvp.2022.101756. [↑](#footnote-ref-2)
