# Supplementary material for: Leveraging and exercising caution with ChatGPT and other generative artificial intelligence tools in environmental psychology research
Source: Front Psychol. 2024 Apr 8;15:1295275. doi: 10.3389/fpsyg.2024.1295275 (PMC11033305; doi:10.3389/fpsyg.2024.1295275)
Supplement: Supplementary file 5 [file Data_Sheet_5.DOCX]

**Supplementary Data Sheet 5**

**Example 5. Planning for a behavioral mapping procedure - steps, tips, logistical checklist, and contingencies**

**Custom instructions^[[1]](#footnote-1)^:**

You are a behavioral scientist who knows the practical issues in field observation, e.g., the non-linear process and emerging issues. We are discussing a plan for observing children's play behavior in a park. Your answers are concise.

**Model**

Gpt-4-1106-preview

**USER**
If I am going to do a behavior mapping in a park for children's play behavior. What may be some key steps from planning to data collection?

**ASSISTANT**

1. **Define Objectives**: Clearly state what you aim to understand about children's play behavior. Are you interested in the types of play, social interactions, frequency, duration, or spatial usage?
2. **Literature Review**: Look into existing research on children's play behavior to inform your study design and ensure you're not duplicating efforts.
3. **Select the Site**: Choose a park or multiple parks that offer a variety of play settings and are accessible for regular observation.
4. **Obtain Permissions**: If necessary, get approval from park authorities and ensure ethical considerations are met, including parental consent if you plan to record identifiable information about children.
5. **Develop a Coding Scheme**: Create categories for different types of play behaviors and interactions you expect to observe. This could include solitary play, physical play, imaginative play, social interactions, etc.
6. **Pilot Study**: Conduct a preliminary observation to test your coding scheme and adjust it as necessary to ensure it captures all relevant behaviors.
7. **Training**: If you have assistants, train them in the observation and coding methods to ensure consistency and reliability in data collection.
8. **Schedule Observations**: Plan a schedule that covers various times of day and weather conditions to get a representative sample of behavior.
9. **Conduct Observations**: Systematically observe the children's play behavior using your coding scheme. This can be done through direct observation, video recording, or both.
10. **Take Notes**: Record contextual information that the coding scheme might not capture, such as environmental factors, unusual events, or emerging patterns.
11. **Review and Reflect**: Regularly review your data and notes to check for consistency and to adapt to any emerging issues or patterns that were not initially considered.
12. **Data Analysis**: Analyze the collected data to identify patterns and draw conclusions about children's play behavior in the park setting.
13. **Report Findings**: Summarize your findings in a report that discusses the implications for park design, child development, or other relevant fields.

**USER**
I want to focus our discussion on steps 1-10 and ignore 11-13. Also, can you combine some steps into phases (which can include several steps)? It's hard to understand 10 steps. Numbering the phases and steps.

**ASSISTANT**
Certainly! Here's a simplified and condensed version of the process, organized into three main phases:

**Phase 1: Planning**

1. **Define Objectives**: Determine the specific aspects of children's play behavior you wish to study.
2. **Literature Review and Site Selection**: Review relevant research and select an appropriate park for observation.
3. **Obtain Permissions**: Secure necessary permissions and address ethical considerations.

**Phase 2: Preparation**
4. **Develop and Pilot Coding Scheme**: Create a system for categorizing observed behaviors and test it in a pilot study.
5. **Training**: If working with a team, train observers in the coding scheme and observational techniques.

**Phase 3: Data Collection**
6. **Schedule Observations**: Plan when and how often observations will take place to capture a diverse range of behaviors.
7. **Conduct Observations**: Systematically observe and record play behaviors using the established coding scheme.
8. **Take Notes**: Document contextual details and any unexpected occurrences during observations.

By structuring the process into these phases, the steps become more manageable and logically grouped, facilitating a smoother research process.

**USER**
What may be some issues and challenges for phase 1?

**ASSISTANT**
**Phase 1: Planning**

During the planning phase, you may encounter several issues and challenges:

1. **Defining Clear Objectives**: It can be challenging to narrow down and specify the study's goals, especially when dealing with complex behaviors like children's play.
2. **Literature Review**: There might be an overwhelming amount of literature to sift through, or conversely, a lack of research on specific aspects of children's play behavior you're interested in.
3. **Site Selection**: Finding a park that offers a representative sample of the population and behaviors you want to study can be difficult. Factors like location, size, facilities, and user demographics must be considered.
4. **Permissions and Ethics**: Obtaining consent from park authorities and parents can be time-consuming. Ensuring anonymity and dealing with ethical issues, such as photographing children or intervening in their activities, can be complex.
5. **Environmental Variability**: Parks are subject to changes in weather, seasons, and time of day, which can influence play behavior and the feasibility of observations.
6. **Resource Limitations**: Budget constraints, limited access to equipment, or personnel can restrict the scope of your study.
7. **Safety and Welfare**: Ensuring the safety of the children observed, as well as the researchers, is paramount and can present logistical challenges.

Addressing these issues in the planning phase is crucial for laying a solid foundation for the study and mitigating potential problems during data collection.

**USER**
Five biggest Challenges for phase 2

**ASSISTANT**
**Phase 2: Preparation**

The following are five significant challenges that may arise during the preparation phase:

1. **Developing an Effective Coding Scheme**: Creating a coding scheme that is comprehensive yet flexible enough to capture the nuances of children's play behavior is challenging. It must be detailed enough to distinguish between different behaviors but also practical for observers to use in real-time.
2. **Pilot Study Limitations**: Conducting a pilot study can reveal unforeseen issues with the coding scheme or observation methods. However, time constraints or limited access to the site may restrict the ability to conduct a thorough pilot study, potentially leading to less reliable data.
3. **Training Consistency**: Ensuring that all observers are trained to apply the coding scheme consistently is critical to the reliability of the data. Different interpretations of behaviors by observers can lead to inconsistent data.
4. **Observer Bias**: Observers may bring their own biases to the data collection process, which can influence their observations and recordings. It is a challenge to minimize these biases through training and the design of the coding scheme.
5. **Logistical Coordination**: If the study involves multiple observers or a large study site, coordinating the logistics of who observes where and when, ensuring adequate coverage of the park, and managing the data collection process can be complex.

Addressing these challenges is vital to ensure that the data collected during the observation phase is valid, reliable, and accurately reflects the children's play behavior.

**USER**
Without prior experience, I am not sure how to start. This is because the first step requires knowing the available parks and the children there. Please let me know some interdependencies in your steps. Then, give me five tips on how to start it.

**ASSISTANT**
Interdependencies in the steps mentioned earlier include:

1. **Objectives and Literature Review**: Your objectives should inform your literature review. The literature review, in turn, can help refine your objectives by highlighting gaps or areas of interest in existing research.
2. **Literature Review and Site Selection**: The literature may suggest certain types of parks or settings that are more conducive to the kinds of play behavior you're interested in observing, influencing your choice of site.
3. **Site Selection and Permissions**: The park you select must not only be suitable for your study in terms of its characteristics and the behaviors it elicits but also in terms of the feasibility of obtaining the necessary permissions for observation.
4. **Objectives and Coding Scheme**: The objectives of your study will directly shape the development of your coding scheme, as it needs to capture the specific behaviors that are relevant to your research questions.
5. **Coding Scheme and Training**: The complexity of your coding scheme will affect the training process; a more complex scheme requires more intensive training to ensure consistency among observers.

Here are five tips on how to start your observational study:

1. **Explore Local Parks**: Visit various local parks to get a sense of the environments and the types of play behavior that occur. This will help you in selecting a site that aligns with your research objectives.
2. **Informal Observations**: Before starting formal observations, spend time informally watching children play to become familiar with the range of behaviors and to start thinking about how to categorize them.
3. **Connect with Community**: Engage with park officials, local community groups, or schools to understand the demographics of children who frequent the parks and to potentially facilitate the permissions process.
4. **Network with Other Researchers**: Reach out to researchers who have conducted similar studies to gain insights into their methodologies and challenges they faced.
5. **Start Small**: Consider conducting a small-scale pilot study or observations at just one park to refine your methodology before expanding the scope of your research. This can help in identifying any issues with your approach early on.

By following these tips and understanding the interdependencies of the steps involved, you can begin your observational study with a more informed and structured approach.

**USER**
What can be some common issues related to the people on site, like they noticed me and influenced me? Please use well-known concepts in research methods so I can further search for them.

**ASSISTANT**
When conducting field observations, several well-known issues related to the presence of the observer and the reactions of people on site may arise. These include:

1. **Hawthorne Effect**: This refers to the phenomenon where individuals modify their behavior in response to their awareness of being observed. Children or even their caregivers in the park might change their behavior because they know they are being watched.
2. **Observer Effect**: Similar to the Hawthorne Effect, the observer effect emphasizes the changes in behavior directly caused by the presence of the observer, regardless of whether the subjects are aware of being observed.
3. **Demand Characteristics**: This concept describes situations where the subjects of a study change their behavior based on their perceptions of what the study is about and what the researchers expect to find.
4. **Reactivity**: This is a broader term that includes any change in behavior by the subjects of observation when they become aware of the observer. Reactivity can encompass both the Hawthorne Effect and demand characteristics.
5. **Observer Bias**: This occurs when the observer's expectations, experiences, or prejudices influence their observations and interpretations of the behavior they are recording.

To mitigate these issues, researchers can:

- Use unobtrusive observation methods where possible, such as behind one-way mirrors or using concealed cameras (with proper ethical approvals).
- Spend time in the field without taking notes or appearing to observe, to acclimatize subjects to the researcher's presence.
- Train observers to be as neutral and non-reactive as possible to minimize their impact on the environment.
- Use multiple observers and cross-check data to reduce the impact of individual observer bias.

Understanding and addressing these issues is crucial for the integrity of the data collected during field observations.

**USER**
In the case of observing children play in parks, what are some approaches to reduce reactivity?

**ASSISTANT**
To reduce reactivity while observing children playing in parks, you can employ several strategies:

1. **Unobtrusive Observation**: Position yourself at a distance or in a location where you can observe without being conspicuous. Use natural features like trees or benches to blend into the environment.
2. **Prolonged Engagement**: Spend extended periods in the park without actively observing or taking notes, so that your presence becomes a normal part of the environment to the children and caregivers.
3. **Indirect Observation**: Use tools like remote cameras or binoculars to observe from a distance, minimizing your direct presence in the children's immediate play area.
4. **Non-interactive Role**: Adopt a passive role in the environment, such as sitting and reading a book, to appear disengaged from the play activities.
5. **Habituation**: Conduct observations over a prolonged period so that regular park-goers get used to your presence and gradually pay less attention to you.
6. **Multiple Observers**: Rotate observers to prevent children and caregivers from becoming too familiar with any one observer, which might influence their behavior.
7. **Invisible Technology**: If ethical and permitted, use small, unnoticeable recording devices that don't draw attention to the observation process.
8. **Naturalistic Observation**: Avoid interacting with the children or influencing their play in any way. If approached, be polite but try to minimize engagement.
9. **Ethical Transparency**: When possible, explain your research to adults in a way that does not influence the children's behavior. Assure them that the children's play is the focus, not the individuals themselves.
10. **Data Triangulation**: Use different methods of data collection (e.g., behavioral mapping, time sampling) to confirm findings without relying solely on direct observation, which might be more reactive.

By implementing these approaches, you can reduce the likelihood that your presence will alter the children's natural play behaviors, thus enhancing the validity of your observations.

**USER**
For my first practice observation in several local parks, can you create a logistic checklist with items in different categories?

**ASSISTANT**
Certainly! Here's a logistic checklist for your first practice observation in local parks, organized into categories:

**Preparation:**

- Review research objectives and hypotheses.
- Develop a preliminary coding scheme for behaviors.
- Prepare observation forms or digital templates.
- Charge and pack any necessary electronic devices (e.g., tablet, audio recorder).
- Ensure you have enough writing materials (notepads, pens, pencils).
- Pack a stopwatch or a device with a timer function for precise timing of behaviors.

**Permissions and Ethics:**

- Obtain any required permissions from park authorities for observation.
- Prepare information sheets for caregivers explaining the study (if approaching them).
- Review ethical guidelines for observing children in public spaces.

**Equipment:**

- Binoculars for distant observations (if needed and appropriate).
- Camera for taking non-identifiable wide shots of the play areas (if allowed).
- Voice recorder for verbal notes (ensure it's unobtrusive).
- Portable chair or mat for sitting (if not available on-site).
- Appropriate clothing for weather conditions and to blend in with the park setting.

**Personal Comfort:**

- Water bottle to stay hydrated.
- Snacks for longer observation periods.
- Sunscreen and hat for protection if outdoors.
- First-aid kit for minor emergencies.

**Travel and Access:**

- Map out the route to the park(s).
- Check park hours and best times for observing play behavior.
- Plan for transportation (public transit, parking if driving, etc.).
- Have a backup plan for alternative parks in case of unforeseen closures or events.

**Data Management:**

- Decide on a system for organizing and labeling collected data.
- Set up a secure method for storing any digital data.
- Establish a routine for backing up data after each observation session.

**Contingencies:**

- Check weather forecasts and have a plan for inclement weather.
- Identify restrooms and sheltered areas within the park.
- Have a communication plan in case of emergencies.

By following this checklist, you can ensure that you are well-prepared for your practice observation sessions in local parks.

**USER**
You mentioned contingencies. Can you propose a longer list of unexpected issues and how can we be prepared or adapted to them?

**ASSISTANT**
Certainly, here's an extended list of potential unexpected issues that might arise during field observations in parks and strategies for preparation and adaptation:

1. **Weather Changes**:
   - **Preparation**: Check the weather forecast and have a plan for different weather conditions (e.g., rain gear, sunscreen, warm clothing).
   - **Adaptation**: Have a flexible schedule to shift observation times if necessary, or use indoor observation points like park pavilions.
2. **Park Closures or Special Events**:
   - **Preparation**: Verify the park's schedule in advance and stay informed about special events that could affect normal play behavior.
   - **Adaptation**: Have alternative parks or observation dates ready as backup options.
3. **Equipment Failure**:
   - **Preparation**: Bring spare batteries, backup recording devices, and extra writing materials.
   - **Adaptation**: If a device fails, switch to manual recording methods or use smartphone apps as temporary replacements.
4. **Disturbances or Safety Concerns**:
   - **Preparation**: Familiarize yourself with park rules, emergency procedures, and the location of safety equipment.
   - **Adaptation**: If a disturbance occurs, move to a safe location and continue observations if possible or reschedule for another time.
5. **Interference from Public**:
   - **Preparation**: Have an information sheet or elevator pitch ready to explain your research to curious onlookers without influencing the subjects.
   - **Adaptation**: If approached, be polite, brief, and maintain focus on the observation task.
6. **Children Leaving or Entering the Park**:
   - **Preparation**: Plan for a time-sampling method that can account for the fluid nature of children's presence.
   - **Adaptation**: Adjust your focus to other subjects or areas of the park if the number of children fluctuates.
7. **Health Issues**:
   - **Preparation**: Bring a first-aid kit, personal medications, and know the location of the nearest medical facility.
   - **Adaptation**: If you or a team member feels unwell, prioritize health and reschedule the observation session.
8. **Data Loss**:
   - **Preparation**: Regularly back up digital data and keep physical records organized and secure.
   - **Adaptation**: If data is lost, use backups and reconstructed notes from memory as soon as possible.
9. **Observer Fatigue**:
   - **Preparation**: Schedule breaks and shorter observation periods to prevent burnout.
   - **Adaptation**: Rotate observers or take a short walk to refresh if feeling fatigued.
10. **Ethical Concerns**:
    - **Preparation**: Review ethical guidelines thoroughly and have a clear protocol for handling sensitive situations.
    - **Adaptation**: If an ethical concern arises, such as a child in distress, follow the protocol, which may include intervening or stopping the observation.

By anticipating these issues and having strategies in place, you can minimize disruptions to your observations and maintain the integrity of your data collection process.

**USER**
You mention "Interference from Public: Preparation: Have an information sheet or elevator pitch ready to explain your research to curious onlookers without influencing the subjects." Can you create a short sheet with potential situations and how to handle them?

**ASSISTANT**
Certainly! Here's a short information sheet with potential situations you might encounter with the public and suggested ways to handle them:

**Observation Study: Children's Play Behavior in Parks**

**Researcher's Brief**: We are conducting a study to understand how children play in park environments. This research will help improve public spaces for children's play and development.

**Potential Situations and Responses**:

1. **Curious Onlooker**: "What are you writing down/observing?"
   - **Response**: "I'm studying how children play and interact in parks to inform better design and facilities for children's play areas. I assure you that all observations are non-intrusive and for research purposes only."
2. **Concerned Parent**: "Why are you watching my child?"
   - **Response**: "I understand your concern. I'm a researcher observing play patterns to contribute to child development studies. I'm not recording any personal information, and your child's privacy is fully respected."
3. **Park Official/Security Inquiry**: "Do you have permission to be doing this?"
   - **Response**: "Yes, I have obtained the necessary permissions to conduct observations here. I can provide documentation if needed and assure you that the study is purely for academic purposes."
4. **Fellow Researcher/Student**: "Are you collecting data for a study? Can you tell me more about it?"
   - **Response**: "Yes, I'm observing play behaviors to learn about the different ways children use park spaces. The findings will contribute to research on child development and urban planning."
5. **Passerby Offering to Help**: "Do you need any assistance with your study?"
   - **Response**: "Thank you for the offer, but the study requires non-participant observation. However, if you're interested in learning more or contributing to future research, I can take your contact details."
6. **Local Community Member**: "How will this benefit our community?"
   - **Response**: "The insights from this study will be shared with park authorities and urban planners to enhance local parks, making them more enjoyable and suitable for children's play."

**General Guidelines for the Researcher**:

- Always be polite and professional.
- Keep explanations simple and clear.
- Respect privacy and avoid sharing specific details about observed individuals.
- If someone appears uncomfortable or asks you to stop observing, comply with their request.
- Carry identification and a copy of the research protocol or permission letter.
- If necessary, direct individuals to your supervisor or the institution overseeing the research for further information.

This information sheet can be tailored to fit the specific context of your study and can be handed out or verbally communicated as appropriate.

**USER**
Now I have an update: I found local parks have fewer children and types of behaviors partly because of the park's design. But a city 2 hours away has a busier and well-designed park. I do not know which parks to choose. Can you propose some questions that are helpful to make the decision (and how does an answer relate to the local park option)?

**ASSISTANT**
When deciding between conducting observations at local parks with fewer children and behaviors or traveling to a busier, well-designed park in a city two hours away, consider the following questions:

1. **What are the primary objectives of your study?**
   - If you aim to study a wide range of behaviors in a dynamic setting, the well-designed park may offer more diversity. However, if the focus is on how park design influences play, the local parks could provide valuable insights into the effects of less optimal designs.
2. **How representative are the parks of the population you wish to study?**
   - If the local parks are not frequented by a diverse group of children, the data may not be generalizable. The busier park might offer a more representative sample.
3. **What logistical constraints do you face?**
   - Consider the time and cost of travel to the distant park. Local parks offer convenience and flexibility for repeated visits, which is especially important for longitudinal or multi-session studies.
4. **What is the scope and scale of your study?**
   - If your study is small-scale or preliminary, starting with local parks might be more manageable. For larger-scale studies, the busier park could provide more comprehensive data.
5. **How does the park setting align with your research questions?**
   - If your research questions are about how children interact in busy, stimulating environments, the city park is more suitable. If they're about play in everyday, possibly less stimulating environments, local parks might be more relevant.
6. **What are the ethical considerations?**
   - Obtaining consent and managing privacy may be easier in local parks where you can build relationships with regular visitors. In a busier park, the sheer volume of visitors could make this more challenging.
7. **What resources are available to you?**
   - Assess whether you have the resources to sustain regular observations at a park that's two hours away. Local parks require fewer resources and may be more practical for a sustained period.
8. **What is the potential for data variability and richness?**
   - A busier park may provide a richer dataset with more variables to analyze. However, local parks might offer the opportunity to observe behaviors in a less complex environment, which could be beneficial for certain research questions.
9. **How will the park choice impact the analysis and conclusions?**
   - The setting will influence the behaviors observed and thus the conclusions you can draw. Ensure that the park you choose aligns with the intended outcomes of your research.
10. **Can the findings from one park inform or complement the other?**
    - Consider whether starting with local parks could inform a subsequent study in the busier park, or vice versa. Sequential studies could provide a more comprehensive understanding of children's play behavior across different settings.

Reflect on these questions and how they relate to your specific research goals. The answers should guide you toward the option that best meets the needs of your study while considering practical and ethical implications.

1. The message that ChatGPT consistently remembers within a chat, see https://openai.com/blog/custom-instructions-for-chatgpt. [↑](#footnote-ref-1)
