# Supplementary material for: Leveraging and exercising caution with ChatGPT and other generative artificial intelligence tools in environmental psychology research
Source: Front Psychol. 2024 Apr 8;15:1295275. doi: 10.3389/fpsyg.2024.1295275 (PMC11033305; doi:10.3389/fpsyg.2024.1295275)
Supplement: Supplementary file 6 [file Data_Sheet_6.DOCX]

**Supplementary Data Sheet 6**

**Example 6 Learning about audiences and stakeholders – Prepare for the interview and communication with the local community.**

**Custom instructions^[[1]](#footnote-1)^:**

You are a community volunteer and communication specialist with experience. You will be required to work on a planning project to assist local planning staff with an environmental psychology study within an urban community. The topic of this study is to explore the role and significance of greenery in this community. Different stakeholders have unique needs that you need to be prepared to coordinate and respond to. Your questions and communication processes need to take into account the feelings and needs of the different parties involved.

**Model**

Gpt-3.5-Turbo

**USER**

The first subject we encountered was the local Forest Service personnel who needed to manage and register all the large trees in this area. But they were severely understaffed. You need to find out what their needs are and what local trees mean to them. Write an outline of an interview question.

**ASSISTANT**

Interview Question:

1. Can you please provide an overview of your role and responsibilities as Forest Service personnel in managing and registering large trees in this area?

2. In your experience, what are the main challenges or obstacles that you face in managing and registering large trees in this community?

3. How does the presence of large trees contribute to the overall environmental quality and beauty of this community, in your opinion?

4. Are there any specific benefits or services that large trees provide to the local ecosystem or community that you find particularly valuable?

5. Can you share any examples of successful management strategies or initiatives that have been implemented to protect and preserve large trees in this area?

6. What resources or support do you currently lack as Forest Service personnel that could enhance your ability to effectively manage and register large trees in this community?

7. How do you envision the role and significance of large trees in this community evolving in the future, and what actions do you think are necessary to ensure their preservation and continued contribution to the community's well-being?

**USER**

The second subject we encountered was a local resident. She is very passionate about nature and has created a small garden inside her yard. Write an outline of an interview question.

**ASSISTANT**

Interview Question:

1. Can you please describe your experience and passion for nature and gardening? What inspired you to create a small garden within your yard?

2. In your opinion, what role does the presence of green spaces and gardens play in the overall well-being of individuals and the community as a whole?

3. How does your small garden contribute to the overall aesthetic appeal and environmental quality of your neighborhood?

4. Can you share any specific benefits or positive experiences that you have personally gained from having a small garden in your yard?

5. Have you encountered any challenges or obstacles in maintaining and nurturing your garden? If so, how have you approached and overcome these challenges?

6. How do you feel your small garden impacts the relationship and interactions you have with your neighbors?

7. In your view, what actions would you suggest to the local community to encourage more individuals to create green spaces or small gardens within their own yards?

8. Lastly, what aspirations or goals do you have for your small garden in the future, and how do you envision it further enhancing your personal connection to nature and the local community?

**USER**

The third subject we met was the husband of the previous lady. While he was very supportive of the garden, he was responsible for most of the physical activity of cleaning and pruning and was, therefore, a bit tired. Please compose an outline.

**ASSISTANT**

Interview Question:

1. Can you please share your perspective on your wife's small garden and the role it plays within your household and the community?

2. How do you personally contribute to the maintenance and physical upkeep of the garden?

3. Could you describe the physical activities involved in cleaning and pruning the garden and the level of effort required?

4. How do you feel about the physical demands of the garden maintenance and the impact it has on your well-being?

5. Are you satisfied with the current division of responsibilities regarding the garden maintenance? Is there any assistance or support that you feel would be helpful in alleviating your fatigue or workload?

6. How do you perceive the benefits of the garden, both in terms of aesthetics and environmental quality, as well as the impact it has on your overall quality of life?

7. Are there any specific strategies or tools that you have found effective in managing the physical demands of garden maintenance?

8. How does the garden contribute to your relationship and connection with nature and the community?

9. In the future, are there any changes or adjustments that you would like to see concerning the garden or its maintenance to ensure a more balanced distribution of physical efforts?

**USER**

The fourth subject we encountered was an adult with asthma. He suffers every year during allergy season because of his pollen allergies. He desperately wanted to get rid of these allergy-causing tree species around his home. Please compose an outline.

**ASSISTANT**

Interview Question:

1. Could you please describe your experience with asthma and the specific challenges you face during allergy season due to pollen allergies?

2. Are there any particular tree species in your neighborhood that you believe to be major contributors to your allergies?

3. How do allergies and asthma impact your daily life and overall well-being, especially during allergy season?

4. Have you taken any specific measures to alleviate your symptoms, such as medications or avoiding certain triggers? If so, how effective have these measures been?

5. Are you aware of any initiatives or strategies undertaken by the community or local authorities to address allergy-causing tree species? If not, what measures would you suggest to mitigate the impact of these trees on individuals with allergies like yourself?

6. In your opinion, what would be the most ideal solution to minimize the presence of these allergy-causing tree species without compromising the overall environmental quality and natural diversity in your community?

7. Have you had any discussions or interactions with your neighbors or local authorities regarding your concerns about allergy-causing tree species? If so, what has been the response so far?

8. Are there any specific resources or support systems that you believe would be helpful in managing your allergies and minimizing exposure to these allergy-causing

**USER**

The fifth subject we encountered was family. They consisted of a couple and three children. One of the older sons was very naughty and once broke his arm climbing a tree. As a result, the couple had a strong grudge against the unmanaged trees in the community and felt that the management organization should regularly cut down the branches to ensure the safety of the children. Please help to write a question outline.

**ASSISTANT**

Interview Question:

1. Could you please share your perspective on the role and importance of trees within the community, especially in terms of the benefits they provide and their contribution to the overall environmental quality?

2. Can you describe the incident where your son broke his arm climbing a tree? How has that experience impacted your perception of tree safety in the community?

3. Do you believe that all trees in the community pose a potential safety risk to children, or are there specific factors or characteristics that make certain trees more hazardous than others?

4. Have you had any discussions or interactions with your neighbors or local authorities regarding your concerns about the unmanaged trees? If so, what has been the response so far?

5. Are you aware of any existing policies or regulations in place regarding tree management and safety within the community? If not, what measures do you believe should be implemented to ensure the safety of children while balancing the benefits of having trees in the community?

6. In your opinion, what would be the ideal approach to balancing the safety concerns surrounding trees while still maintaining the beauty and environmental quality they provide to the community?

7. Have you considered alternative means of ensuring tree safety, such as educating children about safe climbing practices or implementing closer supervision during outdoor

**USER**

The sixth person we met was the HOA president. They want to create a welcoming community through trees, but they have limited funds and manpower. Please help write an outline of the questions

**ASSISTANT**

Interview Questions:

1. Can you please describe the vision and goals of the HOA in creating a welcoming community through trees? How do you believe the presence of trees contributes to creating a welcoming atmosphere?

2. What specific challenges does the HOA face in terms of limited funds and manpower when it comes to tree-related initiatives? How does this impact your ability to achieve your goals?

3. Are there any existing strategies or initiatives that the HOA has implemented or is planning to implement to overcome these challenges and create a welcoming community through trees?

4. How do you prioritize and allocate your limited resources for tree-related projects? What factors do you consider when deciding which tree-related initiatives to pursue?

5. Are there any opportunities for collaboration or partnerships with other organizations or community members to maximize the impact of your tree-related initiatives while minimizing the strain on your resources?

6. In your view, what are the most essential aspects of creating a welcoming community through trees that can be achieved within your current limitations? Are there any specific areas or projects that you believe should be prioritized?

7. How do you communicate and engage with the community regarding tree-related initiatives? What strategies or channels do you utilize to ensure that community members are informed and involved in the process?

8. Have you explored any external funding sources or grants that could potentially support your tree-related initiatives? If not, would you be willing to explore such opportunities to enhance your resources for

**USER**

We need to organize a small focus group research. All the above stakeholders are invited. Please help me write a draft of the organization of the event. And list as many potential conflicts as possible, as well as draft responses.

**ASSISTANT**

Organization of the Event:

Title: "Greenery and Community: A Focus Group Discussion on the Role and Significance of Trees in Our Urban Environment"

Date: [Date]

Time: [Time]

Location: [Venue]

Agenda:

1. Introduction and Welcome: Briefly introduce the purpose of the focus group research and welcome all participants.

2. Icebreaker Activity: Conduct a short icebreaker activity to help participants get to know each other and create a comfortable atmosphere.

3. Presentation: Provide a brief presentation on the importance of greenery in urban environments and the significance of trees in promoting community well-being.

4. Individual Reflection: Allocate time for each participant to reflect individually on their thoughts, experiences, and concerns related to trees and green spaces in the community.

5. Small Group Discussion: Divide participants into small groups and provide discussion prompts related to their specific stakeholders' needs and concerns.

6. Share Insights: Regroup and ask each small group to share the key insights and findings from their discussions.

7. Collaborative Solutions: Facilitate a brainstorming session for all participants to collectively generate potential solutions and strategies to address identified conflicts or concerns.

8. Closing Remarks: Summarize the key points discussed and express appreciation for participants' valuable input and contributions.

**USER**

We would like to prepare to do a workshop to improve understanding. Please help identify any possible stakeholders and design a corresponding recruitment strategy.

**ASSISTANT**

Possible Stakeholders for the Workshop:

1. Local residents

2. Environmental organizations

3. City officials and planning staff

4. Local business owners

5. Parks and recreation department

6. School administrators and teachers

7. Community associations

8. Health professionals and organizations

9. Landscape architects and urban planners

10. Homeowners' associations

Recruitment Strategy:

1. Utilize social media platforms: Create event pages and posts on platforms like Facebook, Twitter, and Instagram to reach a wide audience. Share the event details, purpose, and the benefits of attending.

2. Community bulletin boards and newsletters: Post flyers or announcements in community centers, libraries, neighborhood bulletin boards, and local newsletters.

3. Direct outreach: Contact and collaborate with community associations, local businesses, schools, and other organizations to directly invite their members or patrons to attend the workshop.

4. Email campaign: Send targeted emails to relevant stakeholders, such as city officials, environmental organizations, or local businesses, showcasing the importance of their participation and encouraging them to attend and share the event.

5. Partnerships: Collaborate with local organizations, such as environmental nonprofits or community associations, to co-host or co-promote the workshop, expanding the reach of invitations.

6. Local media: Reach out to local newspapers, radio stations, or TV channels to feature the workshop in community event listings or through interviews. Provide them with a press release detailing the purpose of the workshop and its potential impact on the community.

7. Online event listings: Submit the workshop details to online event platforms in the local area to increase visibility and attract interested participants.

8. Personal networks: Encourage staff members and volunteers involved in the planning to share the workshop details with their personal networks, friends, and family.

Potential Conflicts and Draft Responses:

1. Conflicting schedules: If potential participants have schedule conflicts, consider offering multiple workshop sessions or alternative formats, such as webinars or live-streamed sessions, to accommodate different availability.

2. Lack of awareness or interest: Emphasize the importance of the workshop in addressing community needs and improving the quality of life. Highlight the potential benefits and engage participants through relevant and captivating promotional materials.

3. Skepticism or resistance: Address concerns or skepticism by providing concrete examples of successful cases or studies that highlight the positive impact of greenery in urban environments. Showcase the workshop as an opportunity for dialogue and collaboration to find common ground and actionable solutions.

4. Language barriers: Ensure that promotional materials and communication reach a diverse audience, offering translations or language interpreters during the workshop if necessary.

5. Limited resources: If participants are concerned about workshop costs or resource constraints, highlight any available funding sources or consider seeking support from local sponsors or partners to help offset expenses and ensure the workshop accessibility for all interested parties.

1. The message that ChatGPT consistently remembers within a chat, see https://openai.com/blog/custom-instructions-for-chatgpt. [↑](#footnote-ref-1)
