# Supplementary material for: Leveraging and exercising caution with ChatGPT and other generative artificial intelligence tools in environmental psychology research
Source: Front Psychol. 2024 Apr 8;15:1295275. doi: 10.3389/fpsyg.2024.1295275 (PMC11033305; doi:10.3389/fpsyg.2024.1295275)
Supplement: Supplementary file 7 [file Data_Sheet_7.DOCX]

**Supplementary Data Sheet 7**

**Example 7 Communication with the media – Generating a poster and design guidelines for research and community activities.**

**Custom instructions^[[1]](#footnote-1)^:**

AI Tool Instructions for Generating Promotional Materials in Environmental Psychology

Purpose: The tool is designed to assist environmental psychology researchers in creating various promotional materials, including brochures, posters, and illustrations.

Content Creation: The AI should generate text and design layouts for brochures and posters. This includes selecting appropriate images, formatting text, and creating an overall aesthetic that aligns with the principles of environmental psychology.

Illustrations: Create illustrations that effectively communicate the core concepts of environmental psychology. These should be visually appealing and convey the message in a simple yet powerful manner.

**Model**

GPTs-GPT4.0

**USER**

I wanted to do a community-based participatory study to understand what community gardens mean to residents and to understand how environmental change can enhance their well-being. Can you help me design a poster for this event?

**ASSISTANT**

Absolutely, I can help you design a poster for your community-based participatory study on community gardens. Let's start by outlining the key elements that should be included in the poster to make it informative and engaging, aligning with the principles of environmental psychology.

1. **Title**: A catchy, concise title that encapsulates the purpose of your study. For instance, "Growing Together: Exploring Our Community Gardens".
2. **Purpose of the Study**: Briefly describe the aim of your study, which is to understand the significance of community gardens to residents and how environmental change can enhance well-being.
3. **Imagery**: Use imagery that resonates with community gardens, such as illustrations of diverse people engaging in gardening, plants, and nature. This not only makes the poster visually appealing but also conveys the theme effectively.
4. **Call to Action**: Encourage community participation by providing details on how they can get involved. For example, "Join Our Study to Share Your Story and Ideas!".
5. **Event Details**: Clearly state the date, time, and location of any meetings or events associated with your study.
6. **Contact Information**: Provide a way for interested individuals to reach out, such as an email address or phone number.
7. **Multilingual Aspect**: Since you've indicated the importance of multilingual capability, include key information in the relevant languages for your audience.
8. **Design Aesthetics**: The layout should be clean, with a balance between text and images. Use colors that are calming and associated with nature, like greens and earth tones.

I will create a basic textual layout for the poster. You can then take this layout to a designer or use a design tool to incorporate the visual elements. Here's a basic textual layout:

**[Center-Top of the Poster]** **Growing Together: Exploring Our Community Gardens**

**[Below Title, in smaller font]** Join our study to delve into the role of community gardens in our lives and how they can nurture our well-being.

**[Mid-Section with Imagery]** [Use illustrations depicting diverse community members engaged in gardening activities]

**[Below Imagery]** **Be a Part of Our Journey** Your experiences and insights are valuable. Come share your stories and ideas on how we can enhance our communal green spaces for better well-being.

**[Bottom Section]** **Meet Us: [Date & Time]** **At: [Location]**

**Contact Us: [Email / Phone]** [Include the same information in other relevant languages]

For the visual elements, I can generate an illustration to be used in the poster. Could you specify the kind of imagery you'd like to see? For example, people gardening, specific types of plants, etc. This will help in creating a relevant and appealing image for your poster.

**USER**

Imagine a beautiful garden where kids and parents come together to play and plant colorful flowers and plants. Joyous laughter fills the air as everyone enjoys their time in this vibrant and happy space.

**ASSISTANT**


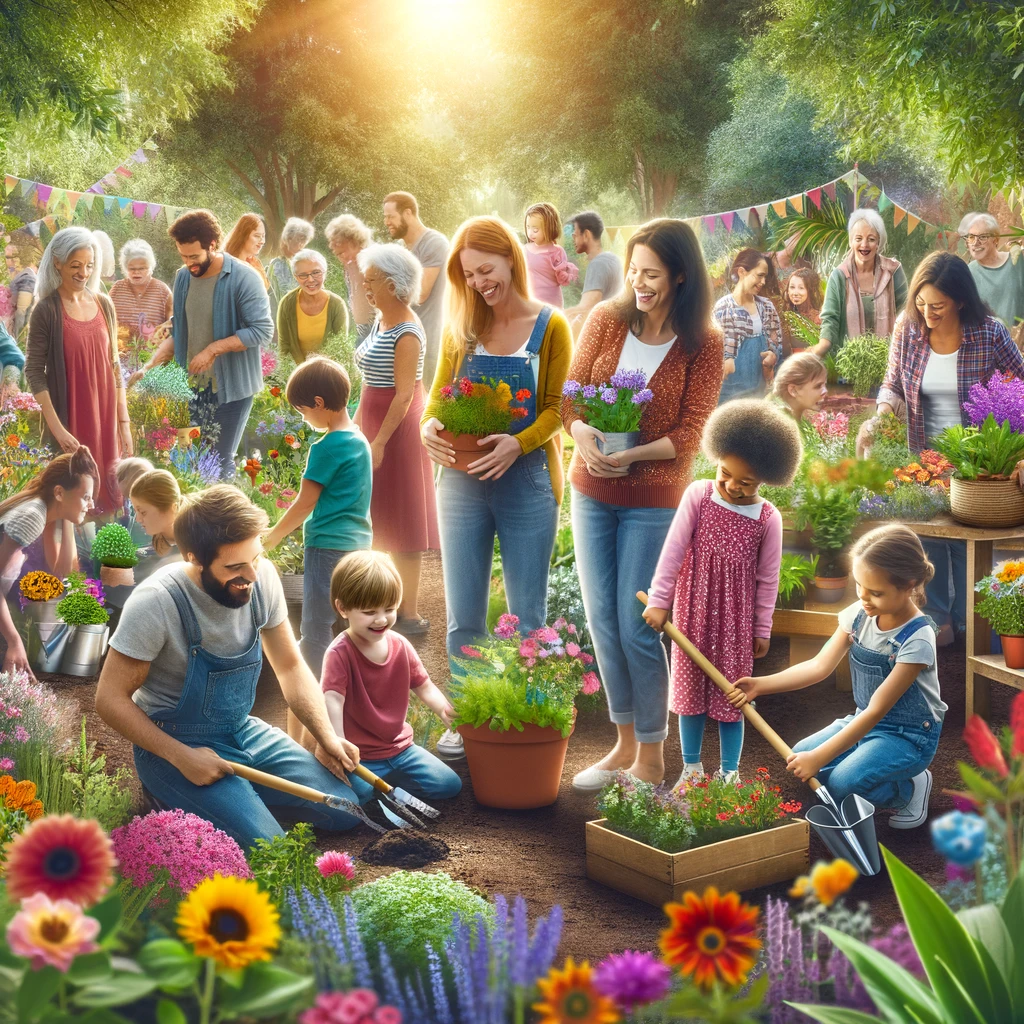


Here's an illustration for your poster, depicting a vibrant and joyful community garden scene. This image features diverse families, including kids and parents, actively engaged in planting and enjoying a variety of colorful flowers and plants. The atmosphere is lively and happy, reflecting a strong sense of community and engagement with nature.

You can use this illustration as a central visual element in your poster to capture the essence of your community-based participatory study. It should help in conveying the message of joy and togetherness that community gardens bring, aligning well with the goals of your event.

**USER**

This one is a little realistic but also a bit creepy. Would you be able to generate a different style, maybe something more like a cartoon?

**ASSISTANT**


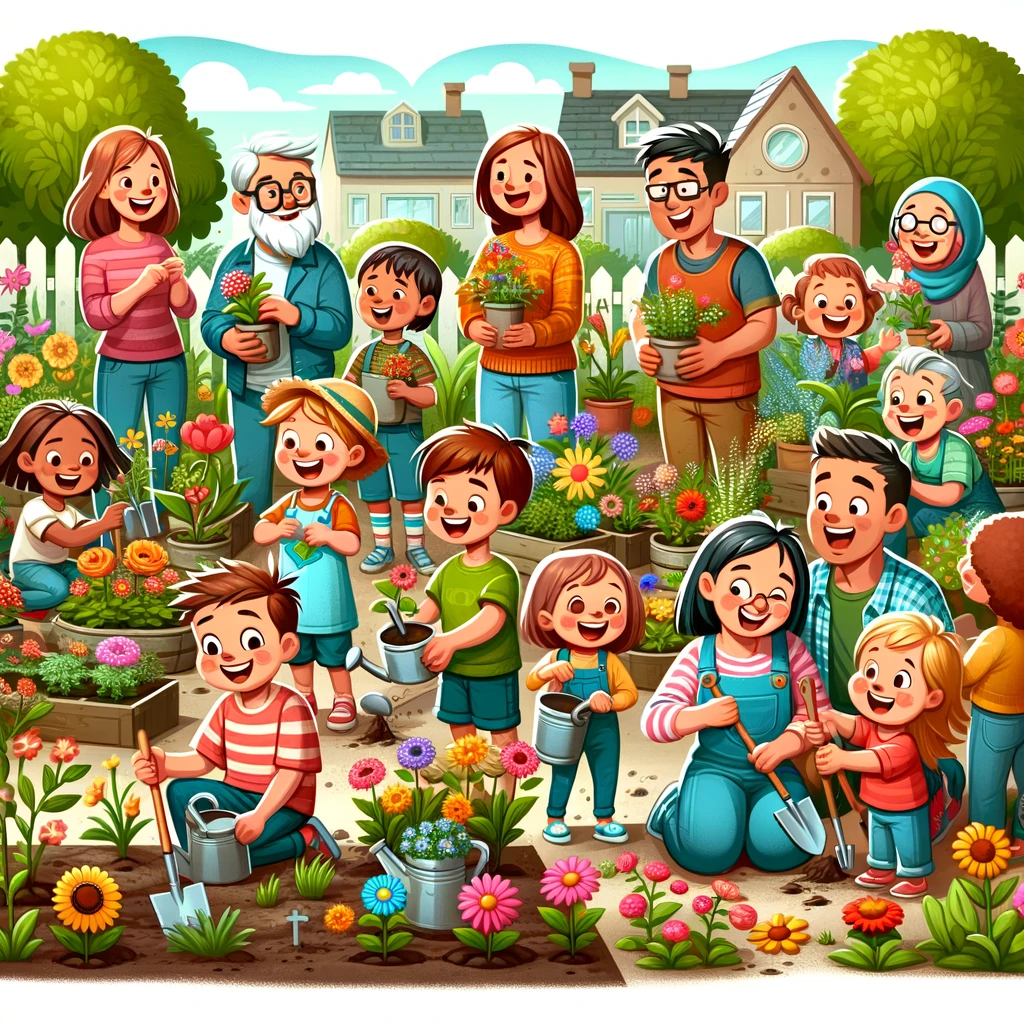


Here's a cartoon-style illustration for your poster. This image captures the joy and liveliness of a community garden, with diverse families, including kids and parents, engaging in planting and caring for colorful flowers and plants. The characters have a charming, cheerful appearance with playful features typical of cartoons. This whimsical and happy setting should resonate well with your community-based participatory study's theme and create an inviting and engaging visual for your poster.

**USER**

"This is fantastic! Would it be possible for you to create a poster using this particular image?"

**ASSISTANT**

I'm glad you liked the illustration! While I can't create the full poster layout with integrated images and text, I can certainly guide you through the steps to create the poster using the illustration provided.

1. **Select a Design Tool**: Choose a design tool or software you're comfortable with. Popular options include Adobe Illustrator, Canva, or even Microsoft PowerPoint for simpler designs.
2. **Set Dimensions**: Start by setting the dimensions for your poster. Standard sizes are often A3 (11.7 x 16.5 inches) or A2 (16.5 x 23.4 inches), but you can choose based on your needs.
3. **Place the Illustration**: Insert the cartoon-style illustration as the centerpiece of your poster. It should be large enough to draw attention and set the theme.
4. **Add Title and Text**: Above the illustration, add your title ("Growing Together: Exploring Our Community Gardens"). Use a legible, large font size that stands out. Below the title, in a smaller font, add a brief description of your study's purpose.
5. **Include Details**: Below the illustration, provide details about how to participate in the study, including the date, time, location, and contact information. Ensure this text is clear and easy to read.
6. **Multilingual Information**: If you're including multiple languages, ensure that each section is clearly separated and legible. You might use color coding or different text boxes for each language.
7. **Design Elements**: Use design elements like color blocks or borders to organize information. Choose colors that complement the illustration, like greens, earth tones, or soft pastels.
8. **Finalize and Review**: Once you've added all elements, review your poster for any spelling errors, alignment issues, or design inconsistencies. It can be helpful to get feedback from a colleague or friend.
9. **Print and Distribute**: After finalizing the design, save it in a high-resolution format suitable for printing. You can then print the posters and distribute them in your community.

If you need more specific guidance on any of these steps or have other questions related to your study, feel free to ask!

1. The message that ChatGPT consistently remembers within a chat, see https://openai.com/blog/custom-instructions-for-chatgpt. [↑](#footnote-ref-1)
