# Supplementary material for: Leveraging and exercising caution with ChatGPT and other generative artificial intelligence tools in environmental psychology research
Source: Front Psychol. 2024 Apr 8;15:1295275. doi: 10.3389/fpsyg.2024.1295275 (PMC11033305; doi:10.3389/fpsyg.2024.1295275)
Supplement: Supplementary file 8 [file Table_1.DOCX]

Supplementary Material

Leveraging and Exercising Caution with ChatGPT and other Generative Artificial Intelligence Tools in Environmental Psychology Research

**Shuai Yuan^1*^, Fu Li^1^, Mondira Bardhan^1^, Matthew H. E. M. Browning^1^, Kuiran Zhang^1^,
Olivia McAnirlin^1^, Muhammad Mainuddin Patwary^2,3^, Aaron Reuben^4^**

^1^ Virtual Reality and Nature Lab, Department of Parks, Recreation and Tourism Management, Clemson University, Clemson, SC 29634, USA

^2^ Environment and Sustainability Research Initiative, Khulna 9208, Bangladesh

^3^ Environmental Science Discipline, Life Science School, Khulna University, Khulna 9208, Bangladesh

^4^ Department of Psychology & Neuroscience, Duke University, Durham, NC USA

*** Correspondence:**Shuai Yuan
syuan2@clemson.edu

# Supplementary Tables

**Table S1.** GAI models and tools classified by their purposes and type of generated content

| **Generated Content** | **Purposes** | **GAI Models and Tools** |
| --- | --- | --- |
| Language and Text | Generate text, dialogue, literature, summaries, or computer program code. | Generative Pre-trained Transformer (GPT) (Brown et al., 2020; OpenAI, 2023)， Open AI ChatGPT^^[[1]](#footnote-1)^^, Microsoft New Bing^^[[2]](#footnote-2)^^, Google Bard^^[[3]](#footnote-3)^^, GitHub Copilot^^[[4]](#footnote-4)^^ |
| Images | Generate images with different genres, realistic levels, and ratio scales. | StyleGAN (Karras et al., 2020), Stability AI Stable Diffusion^^[[5]](#footnote-5)^^, Open AI Dall-E^^[[6]](#footnote-6)^^, Midjourney^^[[7]](#footnote-7)^^, Adobe Firefly^^[[8]](#footnote-8)^^, Skybox AI^^[[9]](#footnote-9)^^ |
| Audio | Generate music composition and audio clips | Singing voice conversion (SVC), Xiaoice X Studio^^[[10]](#footnote-10)^^ |
| Video | Generate video with source material generation, auto-editing, element replacement, animation, and visual effects | Deepfake, Wonder Studio^^[[11]](#footnote-11)^^, Xiaoice Avatar Framework^^[[12]](#footnote-12)^^ |
| 3D Objects | Generate 3D models and environments with point cloud, mesh, color, and textures. | AlphaFold (Tunyasuvunakool et al., 2021), Point E (Nichol et al., 2022), GET3D (Gao et al., n.d.) |

1. https://chat.openai.com/ [↑](#footnote-ref-1)
2. https://www.bing.com/new [↑](#footnote-ref-2)
3. https://bard.google.com/ [↑](#footnote-ref-3)
4. https://copilot.github.com/ [↑](#footnote-ref-4)
5. https://stability.ai/stable-diffusion [↑](#footnote-ref-5)
6. https://openai.com/product/dall-e-2 [↑](#footnote-ref-6)
7. https://www.midjourney.com/home/ [↑](#footnote-ref-7)
8. https://www.adobe.com/sensei/generative-ai/firefly.html [↑](#footnote-ref-8)
9. https://skybox.blockadelabs.com/ [↑](#footnote-ref-9)
10. https://singer.xiaoice.com/ [↑](#footnote-ref-10)
11. https://wonderdynamics.com/ [↑](#footnote-ref-11)
12. https://my.xiaoice.com/ [↑](#footnote-ref-12)
